# Interactive Cognitive-Motor Step Training Improves Cognitive Risk Factors of Falling in Older Adults – A Randomized Controlled Trial

**DOI:** 10.1371/journal.pone.0145161

**Published:** 2015-12-16

**Authors:** Daniel Schoene, Trinidad Valenzuela, Barbara Toson, Kim Delbaere, Connie Severino, Jaime Garcia, Thomas A. Davies, Frances Russell, Stuart T. Smith, Stephen R. Lord

**Affiliations:** 1 Neuroscience Research Australia, University of New South Wales, Sydney, Australia; 2 Institute for Biomedicine of Aging, Friedrich-Alexander University Erlangen-Nürnberg, Nuremberg, Germany; 3 Exercise Science Laboratory, School of Kinesiology, Faculty of Medicine Universidad Finis Terrae, Santiago, Chile; 4 mHealth Laboratory, iNEXT, University of Technology Sydney, Sydney, Australia; 5 RSL LifeCare Chair of Ageing, Australian Catholic University, Sydney, Australia; 6 Faculty of Arts and Business, University of the Sunshine Coast, Sippy Downs, Australia; Ludwig-Maximilian University, GERMANY

## Abstract

**Purpose:**

Interactive cognitive-motor training (ICMT) requires individuals to perform both gross motor movements and complex information processing. This study investigated the effectiveness of ICMT on cognitive functions associated with falls in older adults.

**Methods:**

A single-blinded randomized controlled trial was conducted in community-dwelling older adults (N = 90, mean age 81.5±7) without major cognitive impairment. Participants in the intervention group (IG) played four stepping games that required them to divide attention, inhibit irrelevant stimuli, switch between tasks, rotate objects and make rapid decisions. The recommended minimum dose was three 20-minute sessions per week over a period of 16 weeks unsupervised at home. Participants in the control group (CG) received an evidence-based brochure on fall prevention. Measures of processing speed, attention/executive function (EF), visuo-spatial ability, concerns about falling and depression were assessed before and after the intervention.

**Results:**

Eighty-one participants (90%) attended re-assessment. There were no improvements with respect to the Stroop Stepping Test (primary outcome) in the intervention group. Compared to the CG, the IG improved significantly in measures of processing speed, visuo-spatial ability and concern about falling. Significant interactions were observed for measures of EF and divided attention, indicating group differences varied for different levels of the covariate with larger improvements in IG participants with poorer baseline performance. The interaction for depression showed no change for the IG but an increase in the CG for those with low depressive symptoms at baseline. Additionally, low and high-adherer groups differed in their baseline performance and responded differently to the intervention. Compared to high adherers, low adherers improved more in processing speed and visual scanning while high-adherers improved more in tasks related to EF.

**Conclusions:**

This study shows that unsupervised stepping ICMT led to improvements in specific cognitive functions associated with falls in older people. Low adherers improved in less complex functions while high-adherers improved in EF.

**Trial Registration:**

Australian New Zealand Clinical Trials Registry ACTRN12613000671763

## Introduction

As people age, fluid intelligence and speed-related cognitive performance decline [1]. Recent evidence has also identified impaired higher order cognitive processing as risk factor of falls in older people. Specific cognitive impairments related to poor visuo-spatial skills, impaired attention, reduced executive functioning (EF) and slow processing capabilities can impact the risk of falling in older people [2,3]. There is some evidence suggesting that cognitive functioning can be improved in older people with seated mental training [4] and physical exercise [5]. However, some reviews question the benefits of physical exercise on cognition due to inconsistent findings across studies and methodological issues [6,7]. A further advantage of physical training is its proven effectiveness in reducing fall risk in older individuals [8].

Interactive cognitive-motor training (ICMT) allows task-specific training of various cognitive functions while performing physical exercises. Preliminary findings indicate that such combined physical and cognitive training may lead to larger improvements in cognitive and physical outcomes compared to physical or cognitive training alone [9–12], with possible greater impacts on daily functioning. One type of ICMT is ‘exergaming’ which combines physical exercise with engaging computer games that require multiple cognitive resources. Exergames require players to perform gross motor movements to interact with computer-controlled tasks projected onto a display screen and complex information processing (e.g. dual tasking, inhibiting irrelevant stimuli, decision-making) [13]. Due to its ecological validity, step training may be an efficacious intervention for preventing falls. Repetitive well-timed and directed stepping under cognitive load may lead to motor learning and consequently improve motor programs that can be accessed to initiate steps appropriately in real life [14].

We have developed an inter-active step training system incorporating a modified version of Dance Dance Revolution (DDR) and a choice reaction time task [15] and have demonstrated that eight weeks of unsupervised in-home DDR training improves balance and stepping performance training in older people [16]. The intervention participants also demonstrated a smaller reduction in walking speed under divided attention and a trend for improved performance in a test combining EF and stepping. Three other randomised controlled studies have added modified DDR step training to traditional strength and balance training [17–19]. They reported reduced gait-related dual task costs [18–19] and improved step accuracy under dual task conditions in favor of the stepping interventions [17]. However, to date, it has not been shown that DDR-inspired training alone can improve higher cognitive processing as measured by standard neuropsychological tests (e.g. mental flexibility [16] or working memory [20]).

In the current study, we have augmented our step training system with components that emphasised specific cognitive processing. This was achieved by providing tasks that combined multi-directional stepping at different speeds with mental tasks that accentuated those above mentioned cognitive factors that have been found to be different between fallers and non-fallers. The aim was to assess the effectiveness of this step-based ICMT on cognitive risk factors for falls in older people in a home-based unsupervised setting.

## Materials and Methods

This was a single-blinded parallel-group randomized controlled trial with an allocation ratio of 1:1 (Australian New Zealand Clinical Trials Registry, registration number ACTRN12613000671763, https://www.anzctr.org.au/Trial/Registration/TrialReview.aspx?id=364148).

### Participants

Participants were recruited between June-September 2013 and re-assessments were conducted between September 2013 and January 2014. Individuals were recruited from independent-living apartments of retirement villages in Sydney and from the community. Individuals were eligible if they: i) were aged 70 years or older, ii) lived independently, iii) were able to walk with or without a walking aid, iv) were able to step unassisted on a step pad (step size 25-30cm) and v) had no severe lower extremity pain. Exclusion criteria were major cognitive impairment (Mini-Cog<3) [21], diagnosis of a neuro-degenerative disease, color-blindness, corrected vision of less than 6/16 or an unstable health condition. All participants gave informed written consent prior to study participation. The study protocol was approved by the University of New South Wales’ Human Research Ethics Committee.

### Sample size calculation and randomisation

Data from our previous study [16] was used to estimate the required sample size. We calculated that for an effect size of F = 0.344, a two-sided significance level of 5% and 80% power, a total sample size of 70 participants was required to detect a difference in the Stroop Stepping Task between the intervention and control groups. We anticipated a drop-out of 20% so aimed to recruit 84 people. Permuted block-randomisation using computer-generated random numbers was applied to form two groups of similar size. People living in the same household were treated as one unit and randomized into their own blocks to ensure that equal numbers of couples were allocated to intervention (IG) and control groups (CG). Block size was random and ranged between two and six. The central randomisation office was remote from participant recruitment sites and participant details were provided by email. Screening for eligibility and pre-assessment were undertaken before randomisation.

### Intervention and control content

The interactive training system (described in detail elsewhere [15]) used stepping onto an electronic step pad to interact with a computer interface, and videogame technology was used to deliver the training tasks on standard home television screens. The system was installed in the home of each IG individual and participants were individually instructed how to operate the step training system in a ninety minutes session at the beginning of the trial. The intervention was unsupervised, but to facilitate compliance and to resolve any difficulties with system use, participants were telephoned at the end of weeks 1, 4, 8 and 12. They could also call the research team at other times if required and additional home visits were offered if requested.

**Fig 1:**
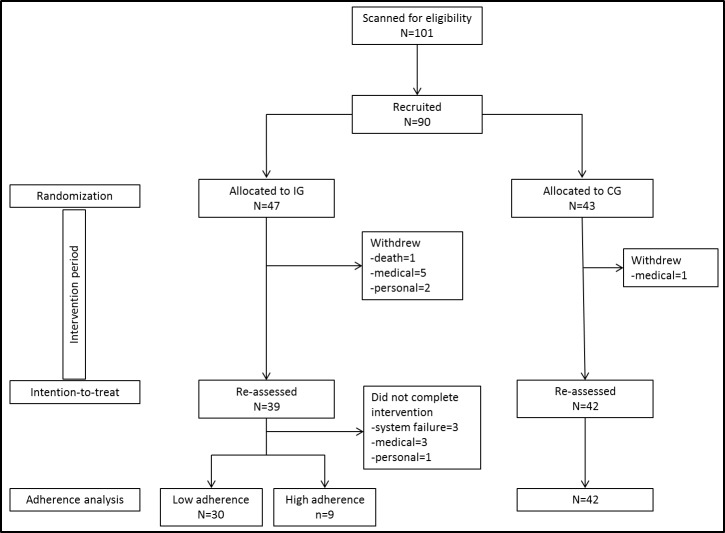
Flow chart of study recruitment and process.

The intervention comprised four games: Stepper, StepMania, Trail-Stepping and Tetris. The basic action of all games entailed making well-timed and directed steps to achieve as many points as possible. In addition, each game also targeted specific cognitive functions associated with fall-risk in older people (Fig 1). We incorporated both, parallel (dual-tasking/multi-tasking) and serial (solve cognitive task before taking a step) processing of stepping and cognition. Individuals therefore were required to maintain their balance under differing postural and cognitive conditions.

*Stepper* comprised a choice reaction task that trained processing speed and visual attention. Individuals viewed a graphical presentation of the mat arrows on the screen. The step direction was indicated by one arrow changing its colour. Participants then stepped as quickly as possible onto the corresponding arrow of the mat and returned to the centre [22]. Levels differed in number of step directions (four vs six) and stimulus presentation time (2s vs 500ms).

*StepMania* trained multi-directional and variable-speed stepping, go/no-go response inhibition and simultaneous processing of multiple stimuli. The objective for this game was to step as accurately as possible in terms of direction and timing. During gameplay, arrows drifted up the screen and when they reached a target arrow position at the top of the screen, participant's had to simultaneously step on the corresponding arrow of the step pad. Our modified DDR game differed from the original version in that we asked participants to return to the centre stance pads after each step. Cognitive load was manipulated by several features. Step responses were not synchronized with the rhythm of the music. Round objects (‘mines’), drifted up the screen in between arrows and for these, participants had to inhibit their step response. In doing so, participants had to selectively attend to some stimuli while inhibiting others; further, this introduced a task of divided attention as they had to perform several processes at the same time. Levels differed in terms of the speed of moving arrows, the number of objects on the screen (distance between objects) and number of distracters (mines).

*Trail-Stepping* trained visual attention and set-shifting. This game comprised a step-version of the Trailmaking test [23]. Participants were required to step on mat panels so as to connect numbers or numbers and letters in alternating order as fast as possible. A representation of the mat was projected on the screen and circles with numbers and/or letters appeared in random order on the panels onto which the participant had to step. To provide participants with feedback about their progress, a red line (trail) was drawn between circles when correct steps were made.

*Tetris* trained visuospatial skills, planning and decision-making. Geometric shapes composed of four square blocks drifted down the screen at a constant speed. By stepping to the left or right, participants moved the shapes on the screen correspondingly. A forward step rotated the shapes and a backward step increased the object drift speed. The objective was to build entire rows/lines in the matrix which would remove them from the screen, creating more space and time to manipulate further drifting shapes and generate game points. The game finished when an unfinished line of shapes reach the top of the screen preventing new shapes from entering. Difficulty levels were increased by increasing the complexity of shapes, the size of the matrix and the drift speed of the blocks.

Each game was played by using the arrows on the step pad, similar to using a keyboard. To ensure an equal number of steps for individuals playing a given game and to avoid multiple small steps, participants were required to return to the two stance panels after each step for all tasks. Participants received visual feedback during game play and after each game as a game score. Each game consisted of a range of levels, with the harder levels requiring higher cognitive capacity and physical effort to perform the tasks. Participants were free to choose which level to play but were encouraged to start the sessions where they had finished last session. They were instructed to progress to a higher level when they considered they were performing well at their current level or considered the game level was not sufficiently challenging, to ensure progression of training intensity. Participants could also return to a lower level if they considered a game level was too difficult. Participants were asked to play each game at least once during each session as many times as they wished with the recommended dose of three 20-minute sessions per week during the 16 week trial. The time and duration for system use and game performance was recorded, saved by the game computer and uploaded to a custom-made website by the system. Participants not using the system for two consecutive weeks were contacted by telephone to encourage adherence.

People allocated to the CG were given a brochure about evidence-based information on various health-related topics, such as fall prevention, staying active, exercising at home, healthy eating, eyesight care, choosing footwear and mobility and walking aids (www.activeandhealthy.nsw.gov.au). CG participants were asked to continue with their usual activities during the study period.

### Outcome measures

Participants underwent the same standardized assessment at baseline and post intervention by trained researchers blinded to group allocation. Socio-demographic and medical information was collected by self-report questionnaires. The 12-item World Health Organization Disability Assessment Schedule (WHODAS) 2.0 was used as a generic assessment instrument of health and disability; participants reported their level of impairment for several instrumental activities of daily living on a five-point Likert scale (www.who.int/classifications/icf/whodasii/en/). To record the number of comorbidities we used the Functional Comorbidity Index for which participants are required to report whether they had one or more of 18 chronic conditions and from which a sum score was calculated [24]. The Mini-Cog was used as screening test to detect major cognitive impairments; it consists of two tasks, delayed recall of three items and the clock drawing test as measure of executive functioning and visuo-spatial ability [21]. Anthropometric measures were obtained during the assessments.

#### Primary outcome measure

The Stroop Stepping Test (SST) is a task of combined stepping and EF. Participants stood on the two stance panels of the step pad. In the centre of the screen an arrow was presented pointing in one of four directions (up, down, left, right). Inside the arrow was a written word indicating a different direction. Participants were required to step to the word and in doing so, selectively attend to one stimulus and inhibit the response indicated by the arrow’s shape. After four practice trials (one for each step direction), the time taken to complete 20 trials, the mean trial time excluding erroneous trials, and the number of errors were measured [25].

#### Secondary outcome measures

Processing speed was assessed with the letter-digit and simple and choice reaction time tests. During the letter-digit test, participants were required to match digits and numbers as quickly as possible. The mean, minimum and maximum times were recorded in ms. Simple hand reaction time in ms (average of 10 trials) was measured using a light stimulus and the depression of switch [26]. For the choice stepping reaction time test (CSRT), participants were required to step as fast as possible onto six target panels in response to arrows pointing to the front, sides and back presented briefly (500ms) and in a random order on a display [22]. The mean stepping response time (36 trials) was subdivided into reaction time from stimulus occurrence to foot lift-off (CSRT-RT) and movement time from lift-off to step-down on the panel (CSRT-MT).

The Trail-making Test (TMT) was used to assess attention and EF (set-shifting) [23]. Participants were required to connect numbers (A) or alternating numbers and letters (B) in ascending order. The ratio score (B/A) was calculated to provide a measure of EF [27]. The attentional network test (ANT) was used to measure the efficiency of neural networks involved in attention (orienting, alerting and executive networks) by combining a cued reaction time paradigm with flanker tasks [28]. Participants were required to determine whether a central arrow that appeared above or below a fixation cross pointed to the left or right. Warning (alerting) and spatial (orienting) cues were combined with flanker tasks (neutral, congruent, incongruent). From these different conditions scores for each network could be calculated. Following a practice block of 24 trials, three blocks of 96 trials were administered with two minutes break in between each block.

The Victoria Stroop task was used to measure executive control by response inhibition [29]. This test requires participants to state a colour under three conditions, while supressing habitual responses related to the conditions. In this study, we only used the number of errors made during the colour-word interference task (condition 3) and the efficiency score of inhibition calculated as the ratio of colour-word interference and colour only tasks (condition 3 / condition 1). A custom-made box with four coloured buttons matching the test colours (gren, red, blue, yellow) was used as input device. Divided attention was assessed using the Timed up and go test (TUG) [30] with a concurrent secondary task. Digit-span backwards (working memory) was chosen as the secondary task because this test can be individualized, i.e. the longest correct sequence of numbers repeated in reverse order while seated was used during the TUG. Finally, a mental rotation task was used to measure visuo-spatial performance. Participants were required to determine whether two-dimensional shapes that were rotated clockwise/anti-clockwise represented the same shape or mirror images [31]. Sixty-four trials were conducted and the time per trial and number of errors were recorded. Digit-letter, Victoria Stroop Task and mental rotation were measured using an open source software (http://pebl.sourceforge.net/) [32].

Depressive symptoms were measured using the nine-item Patient Health Questionnaire (PHQ-9) [33]. Concern about falling was measured by the Icon-FES, an iconographic questionnaire depicting line drawings of a person undertaking a range of simple through to more demanding activities of daily living [34]. For both measures items were scored on a four-level Likert scale and higher scores indicated a higher degree of depressive symptoms or concern.

Adherence was measured using the recorded logs of the system-use. Participants were also asked to fill in monthly calendars indicating the days they used the system and the duration of game-play sessions. For three participants, the computer system failed to accurately record game play and the self-report measure of adherence was used instead. Participants were asked to report adverse events by phone as soon as possible after they occurred and during the regular follow-up phone calls. In addition, individuals provided information on falls using monthly calendars for six months from randomisation. In case of a fall, they filled in a questionnaire describing the circumstances of the fall and were telephoned to obtain additional information.

### Statistical analyses

All clinical outcome variables were tested for normality and non-normally distributed data were normalised via log transformation. Independent t-tests were used to determine between group differences at baseline for continuous variables and Chi square tests were used for categorical variables. Analysis of covariance (ANCOVA) was used to determine between group differences at follow-up with baseline performance of the variable under investigation entered as the covariate. Variables with significant covariate-by-group interactions were explored using the calculation of contrasts and corresponding 95% CI in change scores between IG and CG at different levels of the covariate. Between group differences for falls were analysed using the Chi square test. We also conducted pre-planned analyses to investigate dose-response relationships with post-hoc determined cut-points of adherence to guarantee sufficient group sizes. Assuming that low and high adherers would differ at baseline with respect to some clinical and outcome measures, change scores were used instead of ANCOVAs [35]. However, due to the randomized design of this study ANCOVAs were applied to determine differences between adherence subgroups and the CG Student’s t-tests and Mann-Whitney-U tests were used to investigate differences between intervention sub-groups in time played and difficulty level reached for each game. The Alpha level was set to 5%. Analyses were performed with SPSS (version 22 for Windows, IBM Corp.) and Stata (version 13.1 for Windows, Stata Corp. LP).

## Results

### Participant recruitment, retention and adherence

**Fig 2:**
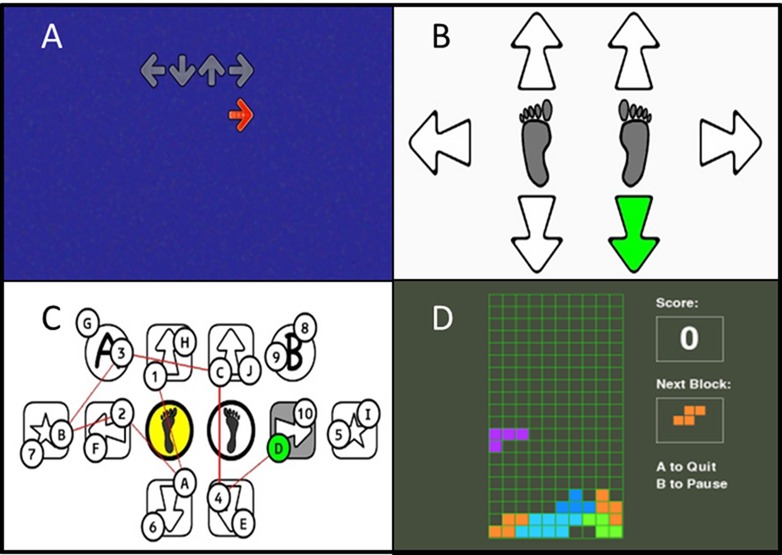
Screenshots of game screens. A–StepMania; B–Stepper; C–Trail-stepping; D–Tetris.

One-hundred-and-one people were screened for eligibility and 90 (IG = 47, CG = 43) met the inclusion criteria and were randomised into the study (Fig 2). The difference in group size was due to the last block in which two couples were allocated to the IG before recruitment stopped. The IG and CG did not differ in any demographic or outcome measure at baseline (Tables 1 and 2). Eighty-one participants (90%; IG = 39, CG = 42) returned for re-assessments after completion of the trial but not all of them were capable of completing all tests. Participants that withdrew during the study period had more co-morbidities (Functional Comorbidity Index p = .030), more difficulties during daily life activities (World Health Organization Disability Assessment Schedule (WHODAS) p = .015), and were more concerned about falling (icon-FES p = .002).

**Table 1:** Sample characteristics.

	Intervention group (n = 47)	Control group (n = 43)	p-value
Age in years	82 ± 7	81 ± 7	0.293
% female	66	67	0.881
Comorbidity (FCI 0–18)	3.55 ± 2.2	2.95 ± 1.8	0.189
Level of disability (WHODAS 0–48)	17.1 ± 5.1	16.1 ± 4.1	0.316
Number medications	4.5 ± 3.3	4.5 ± 3.2	0.962
Depression (PHQ-9)	2.9 ± 4.1	2.1 ± 3.1	0.359
% using a walking aid	28	30	0.782
% who fell in last year	38	28	0.333
Concern about falling (Icon-FES)	53.9 ± 18.2	50.9 ± 16.2	0.411
Overall cognition (Mini-Cog)^^^	4.4 ± 0.8	4.4 ± 0.8	0.940

Note: Data displayed as mean with standard deviation or percentage of individuals; FCI = Functional Comorbidity Index; WHODAS = World Health Organization Disability Assessment Schedule; PHQ-9 = nine-item patient health questionnaire; Icon-FES = Iconographical Fall-Efficacy Scale.

^^^ high score represents better performance.

**Table 2:** Baseline and re-assessment results for outcome measures.

	Intervention Group (N = 39)		Control Group (N = 42)				
	Pre	Post	Pre	post	Adjusted between-group mean diff (95% CI)	p-value baseline	p-value ANCOVA
SST sequence (s)^^^	54.7 ± 15.6	45.5 ± 9.4	61.7 ± 23.2	51.4 ± 15.6	-5.9 (-11.8–-.004)	.170	.217
SST errors	2.49 ± 2.4	1.32 ± 1.7	2.71 ± 2.4	1.29 ± 1.2	.03 (-.62 - .68)	.556	.925
Hand reaction time (ms)^^^	259 ± 45	237 ± 40	261 ± 44	250 ± 40	-12 (-30–5)	.774	**.039**
Digit-letter mean (ms)^^^	2425 ± 374	2274 ± 328	2419 ± 345	2368 ± 334	-93 (-240–53)	.959	**.022**
Digit letter min (ms)	1580 ± 182	1508 ± 162	1601 ± 214	1592 ± 191	-84 (-162–-5)	.642	**.037**
Digit letter max (ms)^^^	4597 ± 2595	4009 ± 1355	4458 ± 2217	4364 ± 1540	-355 (-999–289)	.751	.131
CSRT-RT (ms)^^^	878 ± 126	779 ± 104	925 ± 174	915 ± 233	-136 (-220–-52)	.182	< **.001**
CSRT-MT (ms)	292 ± 67	234 ± 46	314 ± 84	299 ± 84	-65 (-97–-34)	.187	< **.001** ^$^
ANT alert (ms)	34 ± 35	35 ± 33	36 ± 34	32 ± 41	2.7 (-14.2–19.7)	.843	.665
ANT orient (ms)	60 ± 43	57 ± 38	51 ± 46	62 ± 43	-4.7 (-23.3–13.9)	.358	.260
ANT executive (ms)^^^	154 ±120	103 ± 41	145 ± 80	111 ± 45	-6.4 (-26.1–13.3)	.822	.001^$^
TMT A (s)^^^	37.1 ± 19.2	32.8 ± 12.2	38.9 ± 19.1	37.7 ± 14.3	-5.0 (-10.9–1.0)	.618	.*090*
TMT B (s)^^^	110.9 ± 60.0	107.7 ± 47.7	126.8 ± 72.7	128.2 ± 72.8	-20.4 (-48.2–7.4)	.357	.577
TMT B/A (s)	3.1 ± 1.0	3.4 ± 1.1	3.3 ± 1.2	3.4 ± 1.4	-.006 (-.58 - .57)	.404	.406
Stroop CW_ incongruency errors	6.1 ± 4.0	4.7 ± 4.2	6.8 ± 5.8	6.0 ± 4.9	-1.3 (-3.4 - .7)	.506	.273
Stroop efficiency (CW_incongruent/C)	2.2 ± 0.8	1.9 ± 0.7	2.5 ± 1.2	2.2 ± 0.9	-.36 (-.71 - .002)	.183	.150
Dual task (s)^^^	15.9 ± 6.9	15.2 ± 5.0	17.4 ± 7.8	18.5 ± 7.4	-3.3 (-6.2–-.30)	.367	**.030** ^$^
Mental rotation accuracy (%)^#^	78.4 ± 9.9	81.7 ± 11.2	77.8 ± 9.7	74.2 ± 11.6	7.4 (2.3–12.5)	.791	**.001**
Mental rotation time (ms)^^^	5607 ± 2644	5719 ± 3656	6490 ± 3522	5497 ± 2624	221 (-1190–1633)	.200	.*064*
Depression score^^^	2.50 ± 4.20	3.00 ± 4.9	2.12 ± 3.05	3.83 ± 4.1	-.86 (-2.90–1.18)	.589	**.047** ^$^
Icon-FES	50.1 ± 15.7	47.3 ± 13.2	51.1 ± 16.3	53.4 ± 18.3	-6.1 (-13.2–1.0)	.797	**.041**

^#^higher values indicate better performance

^^^log-transformed data were used

^$^sig. co-variate-by-group interaction

**bold** = significant values (p < .05); *italic* = trend for significance (p < .10); IG = intervention group; CG = control group; SST = Stroop Stepping Test; CSRT = Choice Stepping Reaction Time; RT = reaction time; MT = movement time; ANT = Attentional Network Test; TMT = Trailmaking Test (A: time for connecting numbers; B: time for connecting numbers and letters in alternating order; B/A: ratio score of B and A); Icon-FES = Iconographical Fall-Efficacy Scale.

Participants required on average two instruction visits (mean 2.0 (1.2)). During the 16 weeks of intervention, participants played on average 31.8 sessions (SD 21.9) with a mean duration of 27.4 minutes (SD 28.1) for a total of 1317 minutes (SD 2075). Eighteen participants achieved the target of 960 minutes (16 weeks, 3/week, 20 minutes); however, only one participant performed each of the four tasks at least three times per week over 16 weeks. During the trial period, 15 (31.9%) intervention participants withdrew or stopped training (Fig 2). Technical problems with the step training system led to three participants ceasing training and interfered with the training dose of others. One IG participant with post-polio syndrome reported severe leg pain (and subsequent hospitalisation) the day following the instruction session. No falls or other adverse events related to the intervention were reported.

### Effects of the intervention: intention-to-treat analyses

Baseline and re-assessment results including adjusted between-group mean differences for the outcome measures for the intervention groups available for retest are shown in Table 2. No between-group differences were found for the primary outcome measure: Stroop Stepping test time or error scores. There were significant differences favouring the IG for processing speed-related measures: hand reaction time (F_1,78_ = 4.40, p = 0.039), digit-letter mean (F_1,78_ = 5.44, p = 0.022) and minimum time (F_1,78_ = 4.50, p = 0.037) and Choice Stepping Reaction Time-RT (F_1,76_ = 17.57, p<0.001). Additionally, accuracy in mental rotation (F_1,77_ = 11.11, p = .001) and concern about falling (F_1,78_ = 4.34, p = .041) showed improvements compared to the CG. A trend to significance was also evident for TMT-A (F_1_ = 2.939, p = .090).

**Fig 3:**
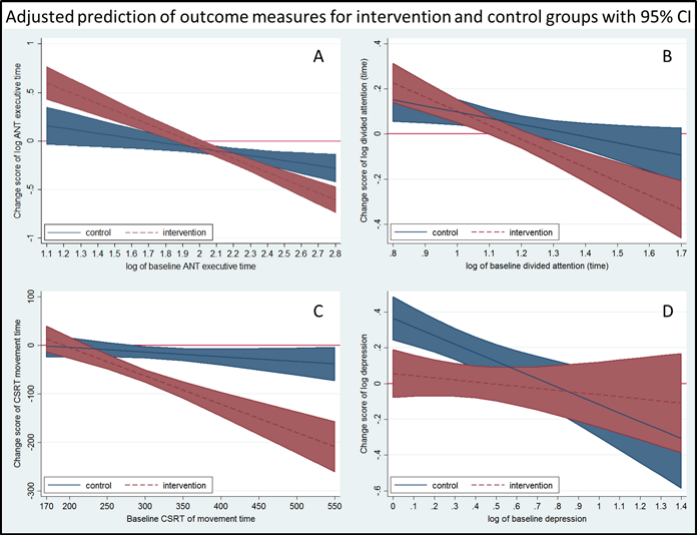
Results interactions. Showing the relationship between baseline scores and change scores at re-assessment; not overlapping confidence intervals (blue and red bands) indicate significant differences between the intervention and control groups.

Further, significant covariate-by-group interactions were observed for tests of ANT_executive (F_1,75_ = 13.161, p = .001), divided attention (F_1,73_ = 4.934, p = .030), CSRT-MT (F_1,76_ = 16.482, p < .001) and depressive symptoms (F_1,76_ = 4.070, p = .047) indicating that group differences varied for different levels of baseline scores. For ANT_executive, IG participants with poorer baseline performance improved more than the CG but for IG participants with better baseline performance, smaller changes were observed (Fig 3A). Similarly, for divided attention and Choice Stepping Reaction Time-MT, IG participants with poorer baseline performance improved significantly more than CG participants (Fig 3B and 3C). Depressive symptom scores did not change as an effect of the intervention, however, CG participants with initial low scores reported higher scores at reassessment compared to the IG in the nine-item Patient Health Questionnaire (Fig 3D). No between group differences were evident for any of the other cognitive outcome measures.

Eighty-four participants provided falls data over the full six months period. Thirteen of the 41 individuals in the IG and 14 of 43 individuals in the CG reported at least one fall (χ^2^ = .007, p = .933).

### Dose-response effects

The IG was divided into those who played three game tasks at least twice per week (high-adherers) and those that did not (low-adherers). High-adherers played all games significantly longer (mean (SD): Stepmania 333 (116) vs 151 (235) min, p < .001; Stepper 119 (49) vs 45 (65) min, p < .001; Tetris 1405 (1509) vs 396 (642) min, p < .001; Trail-Stepping 143 (73) vs 79 (126) min, p < .001). There was no difference in the highest level reached (easy, medium, hard) for each game task. However, high-adherers played higher levels of difficulty more often for Stepper and Trails-Stepping. Low-adherers (n = 30) tended to be older, more concerned about falling, took more medications, had more co-morbidities and performed worse in nearly all outcome measures at baseline than high-adherers (n = 9) (Table 3). Regarding the effectiveness of the intervention, low-adherers improved more in letter-digit mean time and showed trends to higher change scores in the TMT-A test than IG high-adherers, while high-adherers improved more in the ANT_alert and TMT B/A tests and showed trends for larger improvements in Stroop Stepping Test errors than IG low-adherers (Table 4). Complementary results regarding the comparisons between the two adherence groups and the control group with respect to intervention effects are provided in the appendix (S1 File, S1 Fig).

**Table 3:** Baseline comparison between the low and high adherence groups.

	Low adherence (n = 30)	High adherence (n = 9)	p-value
Age in years	83.1 ± 6.7	80.4 ± 7.9	.323
% age 82 or higher	63	33	.142
Comorbidity	3.5 ± 2.3	2.5 ± 1.6	.259
WHODAS	17.1 ± 5.4	14.0 ± 2.9	.106
Number medications	4.7 ± 3.1	2.7 ± 2.2	.*080*
% >4 medications	48	11	.*061*
Depression score^^^	2.9 ± 4.6	1.1 ± 1.8	.129
% depression ≥1	73	38	.*094*
% walking aids	20	33	.406
SST sequence (s)^^^	56.7 ± 17.3	48.1 ± 3.9	**.025**
SST errors^^^	2.6 ± 2.7	2.2 ± 1.0	.529
Icon-FES	51.7 ± 17.0	44.8 ± 9.2	.123
% icon-FES >65	30	0	.*085*
Mini-Cog	4.4 ± 0.8	4.2 ± 0.8	.485
% painful feet	38	0	**.038**
Hand reaction time (ms) ^^^	259 ± 47	259 ± 41	.955
Digit-letter mean (ms) ^^^	2489 ± 389	2213 ± 223	**.036**
Digit letter min (ms)	1609 ± 155	1484 ± 236	.*068*
Digit letter max (ms) ^^^	4973 ± 2851	3343 ± 453	**.019**
CSRT-RT (ms) ^^^	891 ± 138	838 ± 59	.315
CSRT-MT (ms)	296 ± 69	278 ± 60	.493
ANT alert (ms)	39 ± 30	18 ± 49	.132
ANT orient (ms)	63 ± 39	51 ± 58	.512
ANT conflict (ms) ^^^	139 ± 96	206 ± 182	.454
TMT A (s) ^^^	38.5 ± 21.1	32.2 ± 10.0	.448
TMT B (s) ^^^	119.2 ± 63.7	83.0 ± 35.0	.*059*
TMT B/A (s) ^^^	3.2 ± 1.0	2.6 ± 0.8	.115
Stroop error	6.4 ± 4.2	4.9 ± 3.3	.319
Stroop efficiency	2.3 ± 0.8	2.0 ± 0.7	.455
Dual task (s)^^^	16.8 ± 7.2	13.1 ± 4.9	.168
Mental rotation accuracy (%)^#^	78 ± 10	77 ± 10	.753
Mental rotation time (ms) ^^^	5855 ± 2825	4777 ± 1810	.322

Adherence score is based on whether participants played at least three games 32 times during the 16-week intervention period

^#^higher values indicate better performance

^^^log-transformed data were used

**bold** = significant values (p < .05); *italic* = trend for significance (p < .10); WHODAS = WHO Disability Assessment Schedule; SST = Stroop Stepping Test; Icon-FES = Iconographical Fall-Efficacy Scale CSRT = Choice Stepping Reaction Time; RT = reaction time; MT = movement time; ANT = Attentional Network Test; TMT = Trailmaking Test (A: time for connecting numbers; B: time for connecting numbers and letters in alternating order; B/A: ratio score of B and A).

**Table 4:** Differences in program effectiveness based on adherence using change scores.

Change scores	Low adherence (N = 30)	High adherence (N = 9)	p-value
SST sequence (s)^^^	-9.2 ± 11.7	-5.4 ± 4.6	.519
SST errors	-1.0 ± 1.96	-1.6 ± 1.3	.*074*
Hand reaction time (ms)^^^	-22 ± 28	-19 ± 24	.669
Digit-letter mean (ms)^^^	-190 ± 231	-20 ± 154	**.033**
Digit letter min (ms)	-99 ± 199	18 ± 152	.113
Digit letter max (ms)^^^	-803 ± 2731	128 ± 1055	.278
CSRT-RT (ms)^^^	-83 ± 90	-97 ± 53	.542
CSRT-MT (ms)	-60 ± 55	-53 ± 50	.732
ANT alert (ms)	-4 ± 32	21 ± 23	**.043**
ANT orient (ms)	-7 ± 40	8 ± 46	.383
ANT executive (ms)^^^	-35 ± 78	-92 ± 201	.702
TMT A (s)^^^	-6.9 ± 16.4	4.5 ± 15.5	.*096*
TMT B (s)^^^	-1.3 ± 29.0	-9.2 ± 24.8	.250
TMT B/A (s)^^^	0.5 ± 1.1	-0.4 ± 1.2	**.044**
Stroop error	-1.38 ± 3.86	-1.56 ± 2.70	.900
Stroop efficiency	-0.37 ± 0.88	-0.15 ± 0.51	.475
Dual task (s)^^^	-0.7 ± 7.6	1.0 ± 4.5	.403
Mental rotation accuracy (%)^#^	2.5 ± 9.4	5.9 ± 7.1	.325
Mental rotation time (ms)^^^	350 ± 2421	-679 ± 1701	.224
Depression^^^	0.4 ± 2.2	0.4 ± 1.2	.729
Icon-FES	-3.9 ± 12.3	0.8 ± 8.1	.296

Adherence score is based on whether participants played at least three games 32 times during the 16-week intervention period

^#^higher values indicate improvement

^^^log-transformed data were used

**bold** = significant values (p < .05); *italic* = trend for significance (p < .10); SST = Stroop Stepping Test; CSRT = Choice Stepping Reaction Time; RT = reaction time; MT = movement time; ANT = Attentional Network Test; TMT = Trailmaking Test (A: time for connecting numbers; B: time for connecting numbers and letters in alternating order; B/A: ratio score of B and A); Icon-FES = Iconographical Fall-Efficacy Scale.

## Discussion

Video games have been identified as a learning paradigm that facilitates general learning with transfer to unpractised tasks [36]. There is good evidence that videogame play leads to cognitive enhancements in perception, processing speed and executive control in younger people [37]. Although previous ICMT studies in older people have reported aspects of EF can be improved by games using Wii balance board [38], Kinect [39] or cybercycling [40], the overall findings of studies evaluating physical exercise effects on cognitive functioning are inconsistent with non-significant effects in most pooled analyses [6].

With respect to the Stroop Stepping Test, the primary endpoint of this study, no between group differences comparing intervention and control groups were found. We could however, demonstrate that the intervention did improve several specific cognitive functions associated with falls in older people. Interestingly, the observed dose-response relationship and sub-group analyses (Table 3, Fig 3, S1 File, S1 Fig) indicated (i) low adherers were lower functioning people but improved in speed-related functions, reduced their concern about falling and had less depressive symptoms than the CG at reassessment, and (ii) high-adherers were higher functioning people and improved more in complex functions related to EF.

Reduced processing speed has been suggested as a major contributor to cognitive aging [1] and is associated with falls in older people [41,42]. The IG improved their performances in low-level processing (simple reaction time) and in more complex tasks that required higher levels of attention (digit-letter, choice reaction time) suggesting faster central processing after exergaming. With the exception of the Choice Stepping Reaction Time task, participants were required to respond manually (hand reaction time,) or verbally (digit-letter) in these tests. This indicates these changes do not just reflect faster movement time specific to the intervention (leg movements) but also improved general processing with transfers to unlearned tasks. In a previous study, Studenski and colleagues found no improvements for digit-symbol performance in a single group stepping ICMT [20]. Our finding of a significant intervention effect in the digit-letter test may relate to this test comprising an overlearned task without a memory component and therefore a purer measure of processing speed.

Visuo-spatial skills are important for everyday life such as walking through challenging environments requiring visuo-spatial attention and accurate object recognition, and a decline in visuo-spatial abilities has been linked to falls in older people [3,43]. In the current study, visuo-spatial improvements were observed in the intervention group as indicated by increased accuracy in a mental rotation task. However, only the high-adherers concomitantly increased their speed in this task. The lower baseline functional performance and/or the lesser game play in low adherers appear to have led to a prioritization of accuracy over speed for this sub-group. This may also reflect the nature of the intervention that, in general, emphasized precision over speed.

Attention and EF are overlapping constructs that are often measured by the same tests. Performance for TMT-A, a measure of attention improved in low-adherers suggesting that participants improved their strategies to scan for correct information. This task has a lower cognitive load than part B in which participants additionally have to shift their attention between two sets, numbers and letters. Part B has been consistently associated with falls in older people [2], and part A has been shown to be a risk factor for falls in frailer individuals [41]. The ratio score (TMT B/A) is considered a good measure of EF and showed significant improvements in high-adherers compared to low-adherers and the CG. Findings of previous studies regarding the effects of step ICMTs on TMT performance are inconsistent. In one study participants with mixed urinary incontinence were required to switch between pelvic floor muscle training and stepping and improved TMT (B-A) times [44]. In contrast, our previous pilot study using similar step training found no improvements in TMT performance in older people [16]. The addition of Tetris and Step Trails, i.e. two games that specifically targeted EF, to the intervention in the current study appears to have added greater task specificity for cognitive task switching. In fact, every training task in our intervention involved visual search and scanning, which may explain why low-adherers improved on TMT-A.

The improvement in set-shifting ability only for high-adherers, however, suggests that a higher dose of exercise may be necessary for improvements in EF. The high-adherers performed better in some measures of EF at baseline, a finding consistent with previous reports showing EF is a key factor in exercise adherence [45]. It has also been reported that aerobic exercise leads to improvements in EF due to increased pre-frontal cortical activation [46]. As exergaming is of light-to-moderate training intensity for older people [47], it is possible the high-adherers trained sufficiently long per session to induce aerobic training adaptations. Finally, it is likely that simply having a higher dose of an EF demanding exercise will lead to larger EF improvements.

Inhibition, another EF associated with falls, was assessed by a Flanker (ANT_executive) and two computerized Stroop tasks, including our primary outcome measure that included stepping as the response. Both, IG and CG showed reduced times at re-assessment in both Stroop tests with no group differences. The failure to show Stroop test improvements may be explained by the lack of conflict resolution tasks during game play. While choice reaction tasks and go/no-go tasks were included, no conflicting stimuli were presented. However, in comparing low with high-adherers, the latter showed a trend for larger reductions in errors made in the Stroop Stepping Test—a measure that was best in discriminating between fallers and non-fallers in a cross-sectional study [25]. The ANT is based on Posner’s model of attention and reflects three disparate attentional networks. Findings comparing different age groups suggest an age-related decline in alerting and especially in executive networks while no change in the orienting network [48]. Orienting and alerting networks showed no IG-CG differences however, high-adherers improved significantly more than low-adherers in the alerting network suggesting a better level of goal-directed preparedness [49]. Both IG and CG improved their executive networks but the IG did so to a larger extent due to greater improvements in participants with poorer baseline performance, especially the high-adherers. The executive and alerting networks have been shown to partially share activations in frontal and fronto-parietal parts of the brain suggesting our ICMT modified neural circuits responsible for EF [49,50]. Similar results were obtained for divided attention, indicating that IG participants with poorer baseline performance improved their ability to dual task more than the CG. Improvements in dual tasking after exercise programs that included step ICMT have been demonstrated in several studies, supporting task-specific requirements [16–19].

Concern about falling and depressive symptoms are psychological conditions that have been consistently linked to decreased cognitive performance and increased risk of falling [51]. Our ICMT reduced the level of concern about falling in people with higher baseline values and mitigated increases in depressive symptoms observed in the CG. These findings accord with the literature showing that exercise can lead to reduced levels of concern [52]. Exercise is considered an effective strategy for reducing depressive symptoms [53], without conclusive evidence whether this is due to physiologic, psychological or cognitive factors [54].

This study found similar fall rates for IG and CG after six months. This has to be interpreted with caution. The objectives were to determine the effect of ICMT on cognitive functioning. Falls were recorded to measure adverse events during the intervention and to help with sample size calculation for future studies. However, the current trial was neither powered to find significant differences between groups nor were falls monitored for long enough (12 months) to detect differences that might occur after the intervention [55].

The current study has several strengths. It is the first to investigate cognitive changes following a stepping ICMT and the first to report on custom made games developed for older people. Third, although study drop-out was substantial, the remaining sample still comprised the largest published study on stepping ICMT to date. Finally, our study demonstrates both the feasibility and effectiveness of unsupervised in-home exercise training. We also acknowledge certain limitations. The finding of two distinct groups with different characteristics at baseline and in their response to the intervention reduced power for the outcome measures of interest. Second, the combination of both physical and cognitive training components in the one intervention precludes determination of whether both aspects were required or whether one aspect was more beneficial than the other. Third, IG participants did not follow a standardised training protocol due to the unsupervised delivery of the intervention and this likely affected the results. Although we recoded each individual’s step training dose we have not included findings regarding particular games played, in relation to their appeal or effectiveness due to many influencing factors (varying outcomes, duration and levels of difficulty for different games) and the relatively small sample size for such sub-group analyses. Fourth, consistent with previous studies [56], poorer baseline performance was associated with lower adherence to intervention. This might be because mobility impairments make it less feasible or rewarding to participate. In this study, a larger proportion of low-adherers reported painful feet, and low-adherers were also older; a factor likely to influence willingness to use modern technologies [57]. Fifth, some technical issues markedly impacted on training adherence in some IG participants and resulted in larger than anticipated support requirements. Further refinements are therefore necessary before this system could be used outside of a research context. Sixth, in order to increase the power, p-values were not adjusted to the number of comparisons potentially inflating the risk of type I errors. Future studies are required to replicate the results. Finally, as in any study of this kind, unintentional loss of blinding may have occurred. However, participants were reminded not to disclose their group allocation under any circumstances, and therefore we feel confident that the risk of unblinding was kept to a minimum.

In conclusion, the study findings show that unsupervised stepping ICMT led to improvements in specific cognitive functions associated with falls in older people. Two distinct groups were identified that differed at baseline and in their responses to the intervention. Low-adherers improved in basic tasks of processing speed and visual scanning and showed decreased levels of concern about falling whereas high-adherers showed improvements in more complex cognitive functions associated with EF. Future studies should investigate larger samples to determine if improvements translate into reduced fall rates and compare the intervention with traditionally delivered cognitive or physical exercise training. Furthermore, studies exploring the underlying task-specific and/or general mechanisms of those improvements are needed to develop game tasks that are optimal for cognitive training and fall prevention. Future studies should also investigate specific dose-response relationships in subgroups of elderly people.

## Supporting Information

S1 Fig Adherence interaction result. **S**howing the relationship between baseline score and change score at re-assessment for ANT_executive; not overlapping confidence intervals (blue and red bands) indicate significant differences between high adherers and control group.
(TIF)

S1 File Adherence subgroup analyses. (DOCX)

S2 File Consort statement checklist. (DOCX)

S3 File Ethics approved study protocol. (PDF)

S4 File Data_Sample characteristics. (SAV)

S5 File Data_Outcomes. (SAV)
