# Supplementary figures and images for: Interactive Cognitive-Motor Step Training Improves Cognitive Risk Factors of Falling in Older Adults – A Randomized Controlled Trial
Source: PLoS One. 2015 Dec 16;10(12):e0145161. doi: 10.1371/journal.pone.0145161 (PMC4682965; doi:10.1371/journal.pone.0145161)

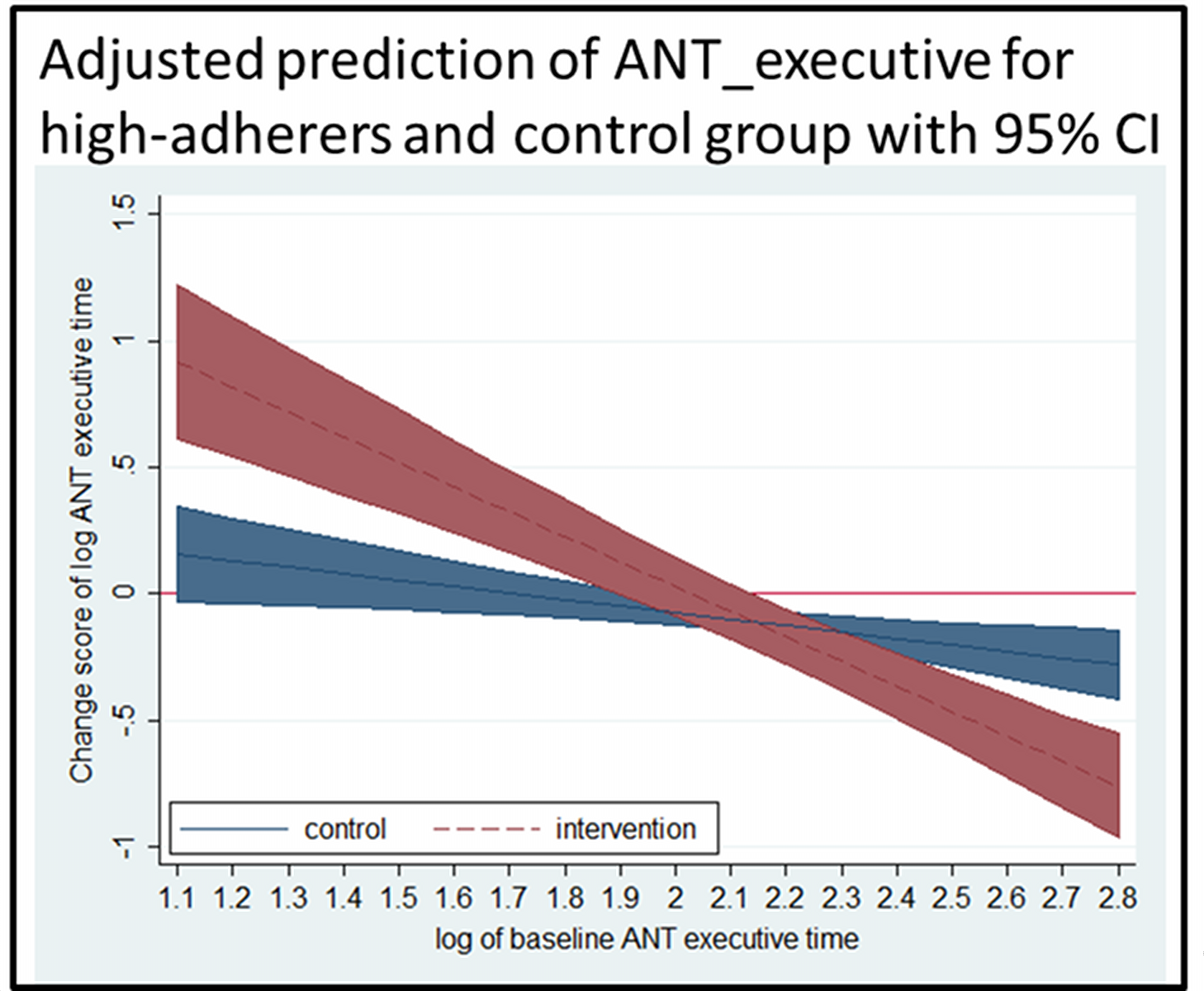

Supplement: S1 Fig — Showing the relationship between baseline score and change score at re-assessment for ANT_executive; not overlapping confidence intervals (blue and red bands) indicate significant differences between high adherers and control group. (TIF) [file pone.0145161.s001.tif]
