# Supplementary material for: Interactive Cognitive-Motor Step Training Improves Cognitive Risk Factors of Falling in Older Adults – A Randomized Controlled Trial
Source: PLoS One. 2015 Dec 16;10(12):e0145161. doi: 10.1371/journal.pone.0145161 (PMC4682965; doi:10.1371/journal.pone.0145161)
Supplement: S1 File — (DOCX) [file pone.0145161.s002.docx]

**S1 File**

**Comparison of low and high adherence groups with the control group**

IG low-adherers improved more than the CG participants in hand reaction time (F_1,69_=4.324, p=.041), digit-letter mean and minimum times (F_1,69_=7.228, p=.009 and F_1,69_=4.724, p=.033 respectively), CSRT reaction and movement times (F_1,66_=12.300, p=.001 and F_1,66_=23.013, p<.001 respectively), TMT-A time (F_1,68_=5.604, p=.021), dual tasking (F_1,63_=4.056, p=.048), and fear of falling (F_1,69_=4.286, p=.042) (Table S1). Additionally the IG low-adherers and CG differed in their depression scores due to an increase in depressive symptoms in the CG (F_1,68_=5.664, p=.020). Mental rotation accuracy improved in the IG (F_1,68_=7.430, p=.008) while the CG improved the time of task accomplishment (F_1,68_=5.799, p=.019).

IG high-adherers improved significantly more than the CG in CSRT reaction and movement times (F_1,48_=9.443, p=.003 and F_1,48_=8.880, p=.005 respectively) and TMT B/A performance (F_1,47_=5.703, p=.021) (Table S1). They also scored better than CG in the mental rotation accuracy task (F_1,47_=6.837, p=.012) while not differing in their speed to do this test (F_1,47_=.055, p=.815). Significant covariate-by-group interactions were found for ANT_executive (F_1,47_=20.539, p<.001) as the IG high adherers were the low performers at baseline, as described above for the whole IG (Figure S1). Effect sizes for hand reaction time (F_1,48_=.782, p=.381) and depression (F_1,46_=3.674, p=.061) were similar for both adherence groups with the non-significant result for the high-adherers likely due to their small sub-group sample size.

Table S1: differences in ANCOVA between intervention group split by adherence (low, high) and control group

|  | Low adherence |  | High adherence |  | Control group |  | Partial eta squared low-CG | Partial eta squared high-CG |
| --- | --- | --- | --- | --- | --- | --- | --- | --- |
|  | Pre | Post | Pre | Post | Pre | Post |  |  |
| SST sequence (s)^^^ | 56.7 ± 17.3 | 46.4 ± 10.4 -18.2% | 48.1 ± 3.9 | 42.6 ± 4.1 -11.4% | 61.7 ± 23.2 | 51.4 ± 15.6 -16.7% | .017 | .014 |
| SST errors^^^ | 2.57 ± 2.7 | 1.54 ± 1.8 -60.1% | 2.22 ± 1.0 | 0.67 ± 0.7 -69.8% | 2.71 ± 2.4 | 1.29 ± 1.2 -52.4% | .005 | .042 |
| Hand reaction time (ms)^^^ | 259 ± 47 | 237 ± 42 -8.5% | 259 ± 41 | 240 ± 34 -7.3% | 261 ± 44 | 250 ± 40 -4.2% | .059* | .016 |
| Digit-letter mean (ms)^^^ | 2489 ± 389 | 2299 ± 349 -7.3% | 2213 ± 223 | 2192 ± 247 -0.5% | 2419 ± 345 | 2368 ± 334 -2.1% | .095** | .000 |
| Digit letter min (ms) | 1609 ± 155 | 1510 ± 154 -6.2% | 1484 ± 236 | 1501 ± 197 +1.1% | 1601 ± 214 | 1592 ± 191 -0.6% | .064* | .007 |
| Digit letter max (ms)^^^ | 4973 ± 2851 | 4170 ± 1387 -16.1% | 3343 ± 453 | 3471 ± 1154 +3.8% | 4458 ± 2217 | 4364 ± 1540 -2.1 | .019 | .036 |
| CSRT-RT (ms)^^^ | 891 ± 138 | 792 ± 112 -11.1% | 838 ± 59 | 741 ± 62 -11.6% | 925 ± 174 | 915 ± 233 -1.1% | .157** | .164^$$^ |
| CSRT-MT (ms) | 296 ± 69 | 236 ± 46 -20.3% | 278 ± 60 | 225 ±48 -19.1% | 314 ± 84 | 299 ± 84 -4.8% | .259** | .156^$$^ |
| ANT alert (ms) | 39 ± 30 | 35 ± 31 -10.3% | 18 ± 49 | 38 ± 39 +111% | 36 ± 34 | 32 ± 41 -11.1% | .000 | .015 |
| ANT orient (ms) | 63 ± 39 | 56 ± 41 -11.1% | 51 ± 58 | 61 ± 29 +19.6% | 51 ± 46 | 62 ± 43 +21.6% | .023 | .001 |
| ANT executive (ms)^^^ | 139 ± 96 | 101 ± 42 -27.3% | 206 ± 182 | 111 ± 40 -46.1% | 145 ± 80 | 111 ± 45 -23.5% | .000 | .324^&^ |
| TMT A (s) ^^^ | 38.5 ± 21.1 | 31.6 ± 10.4 -17.9% | 32.2 ± 10.0 | 36.7 ± 17.0 +14.0% | 38.9 ± 19.1 | 37.7 ± 14.3 -3.1% | .076* | .003 |
| TMT B (s)^^^ | 119.2 ± 63.7 | 117.9 ± 48.7 -1.1% | 83.0 ± 35.0 | 73.8 ± 22.7 -11.1% | 126.8 ± 72.7 | 128.2 ± 72.8 +1.1% | .000 | .048 |
| TMT B/A (s)^^^ | 3.23 ± 1.04 | 3.76 ± .95 -14% | 2.62 ± .84 | 2.19 ± .63 +16.5% | 3.30 ± 1.22 | 3.40 ± 1.42 -3% | .070 | .108^$^ |
| Stroop error | 6.4 ± 4.2 | 5.1 ± 4.4 -20.3% | 4.9 ± 3.3 | 3.3 ± 3.7 -32.7% | 6.8 ± 5.8 | 6.0 ± 4.9 -11.8% | .009 | .027 |
| Stroop efficiency | 2.27 ± 0.84 | 1.90 ± 0.64 -16.3% | 2.03 ± 0.70 | 1.89 ± 0.73 -6.9% | 2.5 ± 1.2 | 2.2 ± 0.9 -12% | .031 | .004 |
| Dual task (s)^^^ | 16.8 ± 7.2 | 15.6 ± 5.5 -7.1% | 13.1 ± 4.9 | 14.2 ± 2.8 +8.4% | 17.4 ± 7.8 | 18.5 ± 7.4 +6.3% | .060* | .014 |
| Mental rotation accuracy (%)^#^ | 78 ± 10 | 81 ± 12 +3.4% | 77 ± 10 | 83 ± 11 +7.8% | 78 ± 10 | 74 ± 12 -5.2% | .099** | .127^$^ |
| Mental rotation time (ms) ^^^ | 5855 ± 2825 | 6205 ± 4013 +6.0% | 4777 ± 1810 | 4098 ± 1128 -14.2% | 6490 ± 3522 | 5497 ± 2624 -15.3% | .079^//^ | .001 |
| Depression | 2.9 ± 4.6 | 3.3 ± 5.4 +13.8% | 1.1 ± 1.8 | 2.0 ± 3.0 +81.8% | 2.12 ± 3.05 | 3.83 ± 4.1 +80.7% | .077* | .074 |
| Icon-FES | 51.7 ± 17.0 | 47.9 ± 13.7 -7.4% | 44.8 ± 9.2 | 45.6 ± 11.5 +1.8% | 51.1 ± 16.3 | 53.4 ± 18.3 +4.5% | .058* | .008 |

^#^higher values indicate better performance; ^^^log-transformed data were used; *p-value<.05 between low adherence and control groups in favour for intervention; **p-value<.01 between low adherence and control groups in favour for intervention; ^//^ p-value<.05 between low adherence and control groups in favour for control; ^$^p-value<.05 between high adherence and control groups in favour for intervention; ^$$^p-value<.01 between high adherence and control groups in favour for intervention; ^&^ significant covariate-by-group interaction
low = low adherence; high = high adherence; SST = Stroop Stepping Test; CSRT = Choice Stepping Reaction Time; RT = reaction time; MT = movement time; ANT = Attentional Network Test; TMT = Trailmaking Test; Icon-FES = Iconographical Fall-Efficacy Scale
