# Supplementary material for: Interactive Cognitive-Motor Step Training Improves Cognitive Risk Factors of Falling in Older Adults – A Randomized Controlled Trial
Source: PLoS One. 2015 Dec 16;10(12):e0145161. doi: 10.1371/journal.pone.0145161 (PMC4682965; doi:10.1371/journal.pone.0145161)
Supplement: S3 File — (PDF) [file pone.0145161.s004.pdf]

THE UNIVERSITY OF
NEW SOUTH WALES

HUMAN RESEARCH ETHICS
COMMITTEE (HREC)

6 February 2009

Dr Stuart Smith
Prince of Wales Medical Research Institute
Barker Street
Randwick NSW 2031

Dear Dr Smith,

Development of a novel intervention for stepping training ability to reduce risk of falls in older adults
HREC 08351

Thank you for your email and attachments to Mrs Annamarie D'Souza dated 2 February 2009.

At the Executive Meeting held on the 3 February 2009 the Committee provided approval for the above project to proceed. In accordance with the guidelines set out in the National Statement on Ethical Conduct in Research Involving Humans* (NS) and exercising the authority delegated by the Deputy Vice-Chancellor (Research), I give permission for this project to proceed.

Would you please note:-

- approval is valid for five years (from the date of the executive meeting i.e. 3 February 2009)
- you will be required to provide annual reports on the study's progress and any adverse events to the HREC, as recommended by the National Statement on Ethical Conduct in Research Involving Humans;
- you are required to immediately report anything which might warrant review of ethical approval of the protocol (NS 2.37), including:

  (a) serious or unexpected adverse effects on participants;
  (b) proposed changes in the protocol; and
  (c) unforeseen events that might affect continued ethical acceptability of the project;

- any modifications to the project must have the prior written approval of the Committee;

UNSW SYDNEY NSW 2052
AUSTRALIA
Telephone: +61 (2) 9385 4234
Facsimile: +61 (2) 9385 6648
Email: ethics.sec@unsw.edu.au
Location: Rupert Myers Building
C/o Research Office / Ethics,
Gate 14, Barker Street Kensington
ABN 57 195 873 179

(08351. cont'd)

- the Ethics Secretariat should be notified if serious or unexpected outcomes are experienced by research participants of if there are unforseen events;

- consent forms are to be retained within the archives of the Institute and made available to the Committee upon request;

- if this approval relates to a clinical trial any serious adverse event arising in the course of the study should be reported promptly using the proforma on the Human Research Ethics website at http://www.ro.unsw.edu.au/ethics/human/

Yours sincerely,

A/Professor Michael Grimm
Presiding Member
HREC

* http:/www.nhmrc.gov.au

UNSW
THE UNIVERSITY OF NEW SOUTH WALES
SYDNEY • AUSTRALIA
HUMAN RESEARCH ETHICS
COMMITTEE (HREC)

28 October 2011

Dr Stuart Smith
Neuroscience Research Australia
Barker St
RANDWICK NSW 2031

Dear Dr Smith

Development of a novel interventions for stepping training ability to reduce risk of falls in older adults
HREC 08351

Thank you for the email and attachment to Mrs Annamarie Dsouza dated 26 May 2011.

The Executive of the Human Research Ethics Committee considered the above request for modification at its meeting held on 31 May 2011 and is pleased to advise it is satisfied that this modification meets the requirements as set out in the National Statement on Ethical Conduct in Human Research*.

Having taken into account the advice of the Committee, the Deputy Vice-Chancellor (Research) has approved this modification to proceed.

Yours sincerely,

Professor Michael Grimm
Presiding Member
HREC

* http:/www.nhmrc.gov.au

UNSW SYDNEY NSW 2052
A U S T R A L I A
Telephone: +61 (2) 9385 4234
Facsimile: +61 (2) 9385 6648
Email: ethics.sec@unsw.edu.au
Location: Rupert Myers Building
Grants Management Office/ Ethics
Gate 14 Barker Street Kensington
ABN 57 195 873 179

08351

ETHICS AND PRIVACY APPLICATION FORM FOR RESEARCH INVOLVING HUMANS

Please Note: Each question on this form has instructions and links to relevant documents and guidelines on how to answer that particular question as hidden text. To show the text with the hidden text effect, click symbol "¶" (**Show/Hide**) (situated next to the **"Zoom"** button) on the "**Standard**" toolbar. When hidden text is shown it is marked with a dotted underline. This text will not be seen on the printed version.

Please note the following:

1. This application must be completed electronically or typewritten
2. Complete all sections except those specifically not applicable
3. Use lay terms wherever possible
4. Do not alter the order of questions or layout of the application form
5. "Y" signifies Yes, "N" signifies No, and "N/A" signifies Not applicable
6. Some "Y"/"N" boxes have been reversed so take care in answering the questions
7. HREC refers to Human Research Ethics Committee

SECTIONS:

		Page
Section 1:	Administration	2
Section 2:	Nature of Research	6
Section 3:	Participants and Recruitment	7
Section 4:	Privacy	10
Section 5:	Collection and Dissemination of Results	13
Section 6:	Risks and Benefits	15
Section 7:	Participant Information and Consent	16
Section 8:	Conflict of Interest and Other Ethical Issues	17
Section 9:	Description of Project	18
Section 10:	Field Based Research or Research Conducted Outside Australia	20
Section 11:	Research Involving Blood, Tissue, etc.	22
Section 12:	Clinical Trials of Drugs or Devices	24
Section 13:	Declaration of Researchers	26
	Checklist	27

This form has been prepared in collaboration between Ms G Briody, Associate Professor M Grimm, Professor A Lloyd, Associate Professor J Watson and Ms M Wright of the Human Research Ethics Committees (HRECs) of the Universities of New South Wales and Sydney.

SECTION 1: ADMINISTRATION

This section is obligatory

1.1 (a) **Full project title**

Development of a novel intervention for stepping training ability to reduce risk of falls in older adults

(b) **Short name by which the project will be known**

Development of a novel intervention for stepping training.

(c) **Name of Chief Investigator**

Dr Stuart Smith

(d) **Provide a brief summary of the project in lay language (approximately 100 words)**

Stepping is often the last protective option to prevent a fall. This study will first modify and validate an interactive system for training stepping ability in older adults. The system will also provide the capability of acquiring indices of stepping ability in the home. We will investigate the effect of an in-home training program using this system on stepping ability and falls risk. Findings will inform future interventions for preventing falls.

(e) **Outline the scientific merits of this study (including potential contributions to the body of knowledge and methodological rigor) (approximately 100 words)**

Falls and fall related injury in older people continue to challenge health and social care systems on a worldwide basis. Studies have demonstrated that impaired stepping is prevalent in older people and that impaired stepping is significantly correlated with high risk of falls. This will be the first study to formally evaluate potential fall-related health benefits of interactive step training system use by older adults. We propose to use existing data acquisition and communication technologies to develop a home-based interactive step training system that will not only aim to improve the stepping ability in older people, but also has the potential to acquire key parameters of this ability in the person's own home.

1.2 **Indicate the institutional ethics committee that you consider to be the primary one for this project. (In general, if the Chief Investigator is a University employee, then the University should be considered to be the primary site. If the Chief Investigator or participants are from a health care service, then the Area Health Service ethics committee should be considered as the primary site.)**

UNSW HREC

1.3 (a) **Has this project already been submitted to any other HREC(s)?** [X] N [ ] Y

(b) **Will this project be submitted to any other HREC(s)?** [X] N [ ] Y

If you answered YES to (a) or (b), give the name of the HREC(s), and indicate the status of the application at each (i.e., submitted, approved, deferred or rejected).
Attach copies of the correspondence with each of the other HREC(s).
Please do not submit to more than one HREC concurrently.

1.4 List the following details of the Chief Investigator/Supervisor, any Co-Researcher(s), Associate Researcher(s) and Student(s).

Chief Investigator/Supervisor

Name	Stuart Smith
Title	Dr
Qualifications	BSc, MSc, PhD
Positions held: employed, conjoint/adjunct/visiting	Senior Research Officer
Full mailing address (including building number)	Prince of Wales Medical Research Institute, Barker St., Randwick, NSW. 2031
Telephone	02 9399 1029
Fax	02 9399 1204
E-mail	s.smith@powmri.edu.au

Co-Researcher(s), Associate Researcher(s), Student(s) or other Personnel involved in the study (If appropriate indicate for each named person whether they are University staff, student or neither). If the named person is a student, nominate (in the Qualifications section) the degree for which he/she is enrolled.

Name	Stephen Lord
Title	Professor
Qualifications	BSc, MA, PhD, DSc
Positions held: employed, conjoint/adjunct/visiting	NHMRC Principal Research Fellow
Full mailing address (including building number)	Prince of Wales Medical Research Institute, Barker St., Randwick, NSW. 2031
Telephone	02 9399 1061
Fax	02 9399 1204
E-mail	s.lord@powmri.edu.au

Name	Catherine Sherrington
Title	Dr
Qualifications	PhD, MPH, BAppSc
Positions held: employed, conjoint/adjunct/visiting	Senior Research Fellow
Full mailing address (including building number)	Musculoskeletal Division, The George Institute for International Health, and Faculty of Medicine The University of Sydney, Level 7, 341 George Street, Sydney, 2000
Telephone	02 9657 0386
Fax	02 9657 0301
E-mail	csherrington@george.org.au

Name	Stephanie Studenski
Title	Professor
Qualifications	PhD
Positions held: employed, conjoint/adjunct/visiting	
Full mailing address (including building number)	Division of Geriatric Medicine<br>3471 Fifth Avenue, Kaufmann Medical Building, Suite 500, Pittsburgh, PA, USA
Telephone	+1 412 692 2360
Fax	+1 412 692 2370
E-mail	studenskis@dom.pitt.edu

Name	
Title	
Qualifications	
Positions held: employed, conjoint/adjunct/visiting	
Full mailing address (including building number)	
Telephone	
Fax	
E-mail	

Insert additional boxes if necessary.

1.5 **Who is the nominated Contact Person (from those listed in 1.4 above) for this protocol?**

Name	Telephone Number	Email
Dr Stuart Smith	02 9399 1029	s.smith@powmri.edu.au

1.6 **Who is the person preparing this document?**

Name	Telephone Number	Email
Dr Stuart Smith	02 9399 1029	s.smith@powmri.edu.au

1.7 **In addition to the researchers named in 1.4 are there students involved as researchers in this project?** [x] N [ ] Y

If you answered YES, indicate the number of students covered by this study and the degrees which this study will contribute towards (i.e., Honours, Masters, PhD, etc.) **If the names are already known please include them.**

1.8 (a) **Indicate the proposed date of commencement of the project.**
Projects may not commence without the prior written approval of the HREC.

Date January 2009

(b) **Indicate the proposed completion date of the project.**

Date December 2011

1.9 **Indicate all location(s) at which the research will be undertaken.**

Prince of Wales Medical Research Institute as well as in the homes of study participants.

1.10 (a) **Has this protocol received research funding/contracting or is this submission being made as part of an application for research funding/contracting?** [ ] N [x] Y

(b) If you answered YES, list the funding/contracting bodies which have been awarded or to which you have submitted, or intend to submit, this project. Attach a copy of the grant application(s), contract(s) or similar agreement(s).

Funding/Contracting body 1: **NHMRC Project Grant 568724**
Funding/Contracting body 2:
Funding/Contracting body 3:

(c) **What is the outcome of these funding/contracting application(s) (please tick the appropriate box)**

	Approved	Pending	Refused
**Funding/Contracting body 1:**	[x]	[ ]	[ ]
**Funding/Contracting body 2:**	[ ]	[ ]	[ ]
**Funding/Contracting body 3:**	[ ]	[ ]	[ ]

(d) **Will this study still be undertaken if funding is not successful?**

N	Y
[ ]	[X]

(e) **If the title of the project submitted for funding is different from that listed under Q1.1(a), state it below.**

Proceed to Section 2.

SECTION 2: NATURE OF RESEARCH
(refer to the National Statement on Ethical Conduct in Research Involving Humans, p. 23-45)

This section is obligatory

2.1 The nature of this project is most appropriately described as research involving:- (more than one may apply):

	N	Y
– behavioural observation	[x]	[ ]
– self-report questionnaire(s)	[x]	[ ]
– interview(s)	[x]	[ ]
– qualitative methodologies (e.g. focus groups)	[x]	[ ]
– psychological experiments	[x]	[ ]
– epidemiological studies	[ ]	[x]
– data linkage studies	[x]	[ ]
– psychiatric or clinical psychology studies	[x]	[ ]
– human physiological investigation(s)	[ ]	[x]
– biomechanical device(s)	[ ]	[x]
– human tissue (see Section 11)	[x]	[ ]
– human genetic analysis (see Section 11)	[x]	[ ]
– a clinical trial of drug(s) or device(s) (see Section 12)	[x]	[ ]
– Other (please specify in the box below)	[ ]	[x]

Participants will be asked to record their use of the stepping training system in an exercise diary. They will be asked to record the time they spend using the system each week as well as provide any free form feedback they may wish to include regarding system use.

Proceed to Section 3.

SECTION 3: PARTICIPANTS AND RECRUITMENT
(refer to the National Statement on Ethical Conduct in Research Involving Humans, p. 25-34)

This section is obligatory

3.1 (a) What is the age range of all participants involved in this study?

**70 years and older**

(b) If the participants include children (defined by statute for this purpose as anyone under 18) has a Prohibited Employment Declaration Form for the researchers ("criminal record check") been lodged with the University or hospital? (see http://www.kids.nsw.gov.au/check/) ☐ Y ☐ N

If you answered NO, give reasons why not.

3.2 Are the participants:-
(more than one may apply)

		N	Y
–	in a teacher–student relationship with the researchers or their associates?	x	
–	in an employer–employee relationship with the researchers or their associates?	x	
–	in any other dependent relationship with the researchers or their associates?	x	
–	wards of the state?	x	
–	prisoners?	x	
–	refugees?	x	
–	members of the armed services?	x	
–	mentally ill?	x	
–	intellectually impaired?	x	
–	unconscious or critically ill patients?	x	
–	under the Guardianship Act 1987 (as amended)?	x	
–	in a doctor–patient relationship or a health giver–receiver relationship with the researchers or their associates?	x	

If you answered YES to any of the above, provide details.

3.3 (a) What is the sample size for the study? Comment on how this sample size will allow the aims of the study to be achieved.

The proposed sample size for the main study is 100 people. Based on our previous experimental studies [3] on voluntary stepping, power analyses (power= 0.8, alpha=0.05) indicate sample sizes of 50 training group and 50 control group will be sufficient for determining clinically significant differences in our outcomes measures. The proposed sample size will also be sufficient for the development of age and sex normative values for persons aged 70 years and older. For the pilot study to develop parameters the step training system software, 20 older and 20 younger adults will be recruited.

(b) How will the participants be recruited?

Participants for the main study will be recruited from older people taking part in a prospective study currently underway and being conducted by Professor Lord [NHMRC Project Grant 510110 Study A, 2008-2010] (separate ethics approval obtained UNSW HREC 07282). We will randomly draw 100 participants (50 women) aged 70+ years from the prospective after their initial assessments of volitional and induced stepping ability, falls risk and additional physiological measures associated with stepping performance such as lower limb power, leaning balance and sensory acuity. The 20 older participants for the pilot study will also be recruited from the prospective study. 20 younger adults will be recruited from staff at the Institute.

3.4 (a) Does recruitment involve a direct personal approach from the researchers to the potential participants? ☐ N ☒ Y

If you answered YES, explain how the real, or perceived, coercion from researchers for potential participants to enrol has been addressed.

Staff from the NHMRC Project Grant 510110 will ask participants if they are willing to participate in the "Novel intervention for training stepping ability" study following completion of Study A of that project. If they agree and are included in the randomization, appointments will be made for the participants to attend POWMRI for the initial step training orientation.

(b) Does recruitment involve the circulation/publication of an advertisement, circular, letter, etc? ☒ N ☐ Y

If you answered YES, provide a copy and indicate where and how often it will be published.

3.5 Will participants receive any reimbursement of out-of-pocket expenses, or financial or other "rewards" as a result of participation? ☐ N ☒ Y

If you answered YES, what is the amount or nature of the reward and the justification for this?

Participants will be reimbursed for any travel expense.

3.6 Is the research targeting any particular ethnic or community group?

[x] N [ ] Y

If you answered YES, which group is being targeted?

If you answered YES, is there an investigator who is a member of the Particular ethnic or community group?

[ ] Y [ ] N

If you answered YES to 3.6, has this project been planned in consultation with a representative of this group?

[ ] Y [ ] N

If you answered YES, who have you consulted and how do they represent this group?

If you answered NO, give reasons why you have not consulted.

Proceed to Section 4.

SECTION 4: PRIVACY
Refer to the National Statement on Ethical Conduct in Research Involving Humans, p. 52-53. For health related information refer to the Statutory Guidelines made under the *Health Records and Information Privacy (HRIP) Act 2002 (NSW) Statutory Guidelines on Research* via Privacy NSW HRIP Act and also the NHMRC overview document *The Regulation of Health Information Privacy in Australia* http://www.nhmrc.gov.au/publications/synopses/nh53syn.htm

This section is obligatory

4.1 Is there a requirement for the researchers to identify, collect, use, or disclose information of a personal nature *(either identifiable or potentially identifiable)* about individuals without their consent?

	N	Y
**(a) from Commonwealth departments or agencies?**	[X]	[ ]
**(b) from State departments or agencies?**	[X]	[ ]
**(c) from other third parties, such as non-government organisations?**	[X]	[ ]

If you answered YES to (a), (b) or (c), state what information will be sought and how many records will be accessed.

4.2 (a) Is there a requirement for the researchers to identify, collect, use, or disclose personal health information about individuals without their consent, which is identifiable or potentially identifiable? [X] N [ ] Y

If you answered NO, you do not need to complete any more of Section 4. Go to Section 5

If you answered YES, indicate the reason(s)

- The project involves linkage of data [ ] Y
- Scientific deficiencies would result if de-identified information was used [ ] Y
- Other [ ] Y

Please provide details

4.3 Will the <u>health</u> information that is identifiable or potentially identifiable with respect to individuals be collected, used or disclosed without the consent of the individual(s) concerned? [x] N [ ] Y

If you answered YES, indicate the reason(s)

- The size of the population involved in the research. [ ] Y
- The proportion of subjects who are likely to have moved or died since the health Information was originally collected. [ ] Y
- The risk of introducing bias into the research, affecting the generalisability and validity of the results. [ ] Y
- The risk of creating additional threats to privacy by having to link information in order to locate and contact subjects to seek their consent of the results. [ ] Y
- The risk of inflicting psychological, social or other harm by contacting subjects with particular conditions in certain circumstances. [ ] Y
- The difficulty of contacting individuals directly when there is no existing or continuing relationship between the organisation and the individuals. [ ] Y
- The difficulty of contacting individuals indirectly through public means, such as advertisement and notices. [ ] Y
- Other [ ] Y

Please provide details

4.4 Was this research the primary purpose of collecting the health information? [ ] Y [ ] N

If you answered YES, you do not need to complete any further questions in Section 4. Go to Section 5

If you answered NO, please provide details

4.5 Would the subjects have expected the researchers to use or disclose their health information for the purposes of this project? [ ] Y [ ] N

Please provide details

4.6 Explain why the collection, use or disclosure of this information is in the public interest, and why the public interest in the project substantially outweighs the public interest in the protection of privacy.

Proceed to Section 5.

SECTION 5: COLLECTION OF DATA AND DISSEMINATION OF RESULTS
(refer to the National Statement on Ethical Conduct in Research Involving Humans, p. 52-53)

This section is obligatory

5.1 **Will any part of the study involve recordings using audio tape, film/video, or other electronic medium ?** [x] N [ ] Y
If you answered YES, what is the medium and how it will be used?

5.2 **Does your research involve the secretive use of photographs, tape-recordings, or any other form of record-taking?** [x] N [ ] Y
If you answered YES, provide details and a justification for the secrecy.

5.3 (a) **How will the results of the study be disseminated (e.g. via publication in journals and presentations in scientific meetings)?**

Publication in peer-reviewed journal articles and conference presentations

(b) **How will feedback be made available to participants (e.g. via a newsletter)?**

At the conclusion of the study, participants will receive a report regarding their performance on a variety of sensorimotor assessments, relative to age-matched norms, in addition to their relative falls risk, and information on evidence-based falls prevention strategies.

5.4 **How will the confidentiality of the data, including the identity of participants, be ensured during collection and dissemination?**

Personal details removed from data prior to entry in computerised database.

5.5 **Is there any possibility that information of a personal nature could be revealed to persons not directly connected with this research?** [x] N [ ] Y
If you answered YES, provide details.

5.6 (a) **What is the proposed storage location of, and access to, materials collected during the study (including files, audiotapes, questionnaires, videotapes, photographs)?**

Paper files will be stored in filing cabinets in locked laboratories. De-identified data will be stored on password-protected computer databases.

(b) **Specify how long materials collected during the study (including files, audiotapes, questionnaires, videotapes, photographs) will be retained after the study, and how they will ultimately be disposed of.**

Please ensure that the period of data retention stated here is appropriate to the nature of the proposed study. If the project involves clinical trial(s), the data should be kept for a minimum of 15 years (please refer to http://www.fda.gov/oc/ohrt/irbs/websites.html). If the projects do not involve clinical trial(s), the data should be kept for a minimum of 7 years after which time the data may be disposed of. *(Please also refer to National Statement on Ethical Conduct in Research Involving Humans, 12.11 for further requirements).*

Data will be kept for 7 years from the end of the study and then disposed by way of a confidential documents bin at the Prince of Wales Medical Research Institute.

Proceed to Section 6.

SECTION 6: RISKS AND BENEFITS (refer to the National Statement on Ethical Conduct in Research Involving Humans, p. 51)

This section is obligatory

6.1 (a) Could participation in the research adversely affect the participants? N [ ] Y [x]

If you answered YES, complete 6.1 (b) and 6.1 (c). If you answered NO go to 6.2

(b) Could the research induce any psychological distress in the participants? N [x] Y [ ]

(c) Could the research cause any physical harm to the participants? (e.g. from physically invasive procedures or from drug administration, etc) N [ ] Y [x]

If you answered YES to (b) or (c) describe the aspect(s) of the research and all the risks involved. Indicate the rate at which these risks are expected to occur. Indicate what facilities and trained personnel are available to deal with such psychological or physical problems.

There is a minimal risk of muscle strain or a fall as a result of the training protocol. However the study protocol will be carried out by experienced research assistants with procedures developed to minimize these risks. Several thousand participants have completed our falls risk assessment without incident. For the in-home step training intervention, use of the step training system will be contingent upon use of a rigid frame surrounding the step mat.

6.2 Will the true purpose of the research be concealed from the participants? N [x] Y [ ]

If you answered YES, outline the rationale and provide details for the concealment. Provide details of the debriefing. (If you do not intend to debrief, give reasons why not).

6.3 Are you doing research on patients (i.e. subjects receiving health care)? N [x] Y [ ]

If you answered YES, list the procedures/techniques which would not form part of routine clinical management.

6.4 Is this research expected to benefit the participants directly or indirectly? N [x] Y [ ]

If you answered YES, provide details.

Proceed to Section 7.

SECTION 7: PARTICIPANT INFORMATION AND CONSENT
(refer to the National Statement on Ethical Conduct in Research Involving Humans, p.12-13, p.28-29, p. 40-42, p.44-45, p.47-50, p.54)

This section is obligatory

7.1 Will a Participant Information Statement be provided? [x] Y [ ] N

7.2 Will written consent be obtained? [x] Y [ ] N

If you answered NO to either 7.1 or 7.2, give reasons why not.

7.3 In the case of participants who may not be fluent in English or who have difficulty understanding English, will arrangements be made to ensure comprehension of the Participant Information Statement and Consent Form? [ ] Y [x] N

If you answered NO, give reasons. If you answered YES, what arrangements have been made?

People who have limited English language skills will be excluded from participation in the study

7.4 (a) Do the Participant Information Statement and Consent Form have:-

- **the first page of the Participant Information Statement and Consent Form printed on appropriate institutional letterhead?** [x] Y [ ] N
- **the title of the project on every page, including the Revocation of Consent? (if one is required) (Use a short title as appropriate)** [x] Y [ ] N
- **the page numbers expressed as page 1 of .., 2 of .., 3 of .. etc?** [x] Y [ ] N
- **an assurance that participation is voluntary and participants are permitted to withdraw from the project at any time without penalty?** [x] Y [ ] N
- **the name and telephone number of an appropriate researcher?** [x] Y [ ] N
- **a telephone number, fax number and E-mail address for the HREC, should a participant wish to make a complaint about the conduct of the research project?** [x] Y [ ] N

(b) How has the possibility of withdrawal from the study been addressed in the Participant Information Statement and Consent Form?

The participant information statement and the consent form state that subjects are permitted to withdraw from the project at any time without penalty or prejudice

Proceed to Section 8.

SECTION 8: CONFLICT OF INTEREST AND OTHER ETHICAL ISSUES
(refer to the National Statement on Ethical Conduct in Research Involving Humans, p. 51–54, Appendix 2)

This section is obligatory

8.1 Are any "conflict of interest" issues likely to arise in relation to this research? [X] N [ ] Y

If you answered YES, provide details.

8.2 Do the researchers have any affiliation with, or financial involvement in, any organisation or entity with direct or indirect interests in the subject matter or materials of this research? [X] N [ ] Y

(Note that such benefits must be declared in the Participant Information Statement.)
If you answered YES, provide details.

8.3 Do the researchers expect to obtain any direct or indirect financial or other benefits from conducting this research? [X] N [ ] Y

(Note that such benefits must be declared in the Participant Information Statement.)
If you answered YES, provide details.

8.4 (a) Have conditions already been imposed upon the use (eg. publication), or ownership of the results (eg. scientific presentations) or materials (eg. audio-recordings), by any party other than the listed researchers? [X] N [ ] Y

(b) Are such conditions likely to be imposed in the future? [X] N [ ] Y

If you answered YES to (a) or (b), provide details.

Proceed to Section 9.

SECTION 9: DESCRIPTION OF PROJECT
(refer to the National Statement on Ethical Conduct in Research Involving Humans, p. 13)

This section is obligatory

9.1 Describe the project using lay terms wherever possible, including the aims, hypotheses, research plan and potential significance. Where relevant, provide the projected number, sex, and age range of participants (including inclusion/exclusion criteria). You must satisfy the HREC that the study is scientifically valid and conducted in accordance with the accepted ethical principles governing research involving humans.
The description must be no longer than 2 pages and must be in a font size of at least 10 points.

Aims:
- To develop a home-based step training system that will reduce the risk of falls
- To determine the effects of a step training intervention on protective and volitional stepping and key indicators of fall risk.
- To establish predictors of adherence to the program.

Design: This study will involve installation of our step training system into the homes of older adults for an investigation of the potential benefits of a home-based program of step training. Older adults will be randomised into either a step training or control group. We will assess whether a 24-week period of training using the system has a significant impact on stepping ability and fall risk.

Participants: will randomly draw 120 participants (60 women) from the prospective study described above (NHRMC Project Grant 510110). 20 younger adults will be recruited from staff and students at the Institute. 20 older adults will participate in Study 1, 100 in Study 2.

(Pilot) Study 1. Step training system development: Interactive, user input devices such as dance pads (Fig 1) are ideally suited for development of a low-cost, interactive method for training stepping ability: "Dance pad" games, where repetitive medio-lateral and anterior-posterior steps are required in response to visual stimuli presented on a video screen offer a novel, yet effective, technique for training stepping ability in older adults. In Study 1 we will develop a dance mat video game that is appropriate for the functional level and interests of older adults. During 6 sessions (1hour/week for 6 weeks), 20 older adults will visit the laboratory at POWMRI and participate in a series of short trials (3 minutes each) that assess stepping ability using a simple computer program that varies the step rate and complexity. Participants will make single steps in response to visual instructions presented on a computer screen. The outcome of this pilot study will establish those parameters of step response that are possible for older adults as well as deliver a step training system that can be deployed into the homes of Study 2 participants.

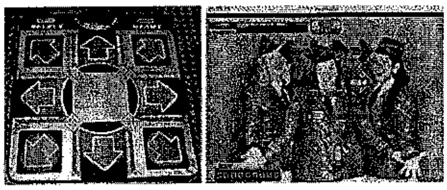

Figure 1. Standard dance pad input device (left) for interacting with dance-style video games such as StepMania (right).

Study 2. Home-based RCT to assess the effect of step training on fall risk. In this study we will install the step training system developed in Study 1 into the homes of 50 older adults who will be randomly assigned into the intervention group. We will assess whether a 24week period of training using the system has a significant impact on stepping ability and fall risk.

Outcome measures: All assessments will be conduced immediately before and at the end of the 24 week trial will be conducted at a laboratory at POWMRI. Choice stepping reaction time, waist pull perturbation responses and PPA fall risk index scores will comprise the primary outcome measures.

Choice stepping reaction time - a composite assessment of balance and reaction time, which has previously been found to be a good predictor of falls in older adults [1]. This measure will provide information regarding individual's volitional stepping performance. Total CSRT (composed of initiation and transfer reaction times) will comprise the primary CSRT outcome measure.

Waist pull perturbation - Participants will be pulled unexpectedly in different directions, with a randomly presented series of 3 forces, 3 displacements and 3 velocities. Participants stand with four very light but stiff cords attached to a broad belt that is fixed securely about the pelvis (Fig 2). The cords have an in-series force transducer (z), connected to a tensiometer mounted on a linear motor (M), so that the subject can be pulled forwards, backwards and laterally. Subjects are told to stand "at ease" and not voluntarily intervene. Movements (displacements, velocities, accelerations) in response to force-controlled perturbations, in addition to the forces underfoot and muscle activity responsible for these observed movements, are quantified. Other outcomes that can be measured using this technique include COM position at step initiation, COM peak velocity, step time, step position, number of steps taken and whether any foot collisions occur in response to lateral perturbations. The primary outcome measure from the waist-pull perturbation test will be the force threshold for stepping.

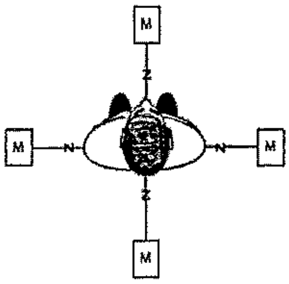

Figure 2. Four linear motor setup for AP and ML waist pull perturbations

Falls Risk - Physiological profile assessment (PPA) - Falls Risk will be determined using the short-form PPA which contains five validated measures of physiological function [2]. In multivariate models, weighted contributions from these five variables provide a falls risk score (primary outcome) that can predict community-dwelling people at risk of multiple falling with 75% accuracy in a 12-month period. The five short-form PPA tests are:

1. Visual Contrast Sensitivity - assessed using the Melbourne Edge Test.
2. Proprioception - measured using a lower limb-matching task. Errors in degrees are recorded using a protractor inscribed on a vertical clear acrylic sheet placed between the legs.
3. Quadriceps strength - measured isometrically in the dominant leg, while participants are seated with the hip and knee flexed to 90 degrees.
4. Simple reaction time - measured using a light stimulus and a finger-press as the response.
5. Postural sway (maximal anterior-posterior and lateral displacement) - measured using a sway meter recording displacements of the body at the level of the pelvis. Testing is performed standing on a foam rubber mat with eyes open.

Randomisation: Participants will be randomised into either the step training or control group using a computer generated random number schedule with randomly permuted block sizes of 6–10. Randomisation will be done centrally by Dr Sherrington who will not be directly involved in participant recruitment (i.e. a concealed randomisation system). There will be 50 participants per group.

Intervention: Following recruitment and randomisation, a home visit to each participant in the training group will be conducted to establish an appropriate location within the home for training and address any possible physical or technical impediments to system use. A purpose-built rigid frame to surround the dance pad will be supplied as a safety measure. During this first home visit participants in the training group will receive a lesson on system use and will be asked to suggest any song titles or visual images (e.g. pictures of grandchildren) they would like incorporated into their personalised step training system. By encouraging an active role by older adults in development of the system, we aim to further promote adherence to exercise training programs.

Home visits by an exercise trainer will be conducted in weeks 1, 2, 3,6 and 12 to encourage ongoing system use, monitor system operation and to discuss any issues which have arisen during previous step training sessions, advise on progression of the step training routines and encourage adherence. Follow-up phone calls to participants will be made monthly during the 24 week study period to encourage ongoing participation in the program rates.

During the intervention, participants will self-select the rate of progression of difficulty and may repeat lessons / songs to improve their "grade" or try new ones. Participants in the training group will be encouraged to engage in at least three 30 minute sessions per week for each of the twenty four weeks of the intervention with a recommended goal of at least 4 sessions and 120 minutes per week of system use. The intensity and type of the step training exercises can be adjusted as performance improves to ensure that the intervention remains challenging.

Adherence, Progression and Adverse Events: Using the interactive step training system deployed into the homes of participants in the step training group, we will also be able to monitor the progression of reaction time indices recorded by the system software. The step training system will also be able to record date and time of use as well as scores of the participants. Adherence rates will be monitored by home exercise diaries kept by participants and compared against usage data automatically recorded by the step training system. Participants will also be asked to identify reasons for adoption and adherence as well as non-adoption and non-adherence. These data will be analysed in a similar manner to the one used in a previous study by Prof Lord [3]. These data will be used to measure adherence to the step training intervention. We will monitor any adverse events due to system use such as falls or sore muscles.

Control group: Participants in the control group will receive usual care so will not be disadvantaged by being in the study. In addition, they will receive an education booklet about falls prevention.

References:

[1] Lord SL and Fitzpatrick RC. 2001. Choice stepping reaction time: A composite measure of falls risk in older people. Journal of Gerontology: Medical Sciences. 56A(10), M627-632

[2] Lord SR, Menz HB et al. 2003. A physiological profile approach to falls risk assessment and prevention. Phys Ther, 83(3), 237-252

[3] Williams, P, Lord SR: Predictors of adherence to a structured exercise program for older women. Psych & Aging. 1995; 10(4): 617-24

9.1 (continued)

Proceed to section 10.

SECTION 10: FIELD-BASED RESEARCH (i.e., CONDUCTED OFF CAMPUS OR OUTSIDE A HEALTH SERVICE) INCLUDING RESEARCH CONDUCTED OUTSIDE AUSTRALIA
(refer to the National Statement on Ethical Conduct in Research Involving Humans, p.14, p.31-32)

This section must be completed for all applications involving EITHER field-based research OR research to be carried out in countries outside Australia (eg. in a school, a corporation, a government department an Aboriginal and Torres Strait Islander community or research in a another country).

10.1 Does this section apply to your research? ☐ N ☒ Y

If NO, Go to Section 11

10.2 Have you obtained formal permission from relevant authorities for entry to the area to carry out research (e. g., national or local government bodies, organisations of local communities)? ☐ Y ☒ N

If you answered YES, name the relevant authorities and attach the relevant correspondence.

If you answered NO, give reasons.

Research will be conducted in the homes of participants. Permission for the research will be obtained from the participants themselves.

10.3 If research is proposed among members of specific organisations, have you sought approval from those organisations (e. g., church groups, national associations, etc)? ☐ Y ☒ N

If you answered YES, name the relevant authorities and attach the relevant correspondence or letter of support.

NA

If you answered NO, give reasons.

10.4 Does the research involve individuals or groups of people who are not formally organised (e.g., people living in a village or town, etc)? ☒ N ☐ Y

If you answered YES, indicate the context of the research. How will you obtain access to participants? Indicate any ethical issues that you can foresee in this approach.

10.5 Will your research necessarily involve the acquisition of objects of valuable cultural property (e. g., carvings, paintings, etc)?

[x] N [ ] Y

If you answered YES, give details of arrangements with owners of the property with regard to access to/acquisition of these items, where appropriate.

10.6 Will your research necessarily involve any activities that are likely to be seen by research participants and/or members of their local communities as in conflict with local practices and customs (e.g. regarding religious or ritual participation)?

[x] N [ ] Y

If you answered YES, provide details.

Proceed to Section 11.

SECTION 11: RESEARCH INVOLVING BLOOD, TISSUE, ETC.
(refer to the National Statement on Ethical Conduct in Research Involving Humans, p.33, p.43-50)

This section must be completed for all research involving blood or tissue samples, or involving physical hazards.

11.1 Does this section apply to your research? [x] N [ ] Y

If NO, Go to Section 12

11.2 Will human blood or tissue be used in the research? [ ] N [ ] Y

If you answered YES, what procedures are in place to minimise the infectious and other risks to participants and researchers?

11.3 Will human embryos, fetal tissue, or placental tissue be involved? [ ] N [ ] Y

If you answered YES, provide details.

11.4 Has this blood or tissue already been collected and stored? [ ] N [ ] Y

If you answered YES, what was the original purpose of collection for the stored blood or tissue you seek to use?

**11.5 Describe the proposed storage arrangements of the blood and/or tissue samples collected.
Indicate how long the blood or tissue will be kept.
Indicate how the samples will be disposed of upon the completion of the research.**

11.6 Will genetically modified organisms or other gene modification techniques be used in the research? [ ] N [ ] Y

If you answered YES, provide details. Describe the procedures, which are in place to minimise the risks to participants and researchers.

11.7 Will toxins, mutagens, teratogens or carcinogens be used? ☐ N ☐ Y

If you answered YES, provide details. Describe the procedures, which are in place to minimise the risks to participants and researchers.

11.8 Will biohazardous material be used? ☐ N ☐ Y

If you answered YES, provide details. Describe the procedures, which are in place to minimise the risks to participants and researchers.

11.9 Will participants or researchers be exposed to ionising radiation? ☐ N ☐ Y

If you answered YES, provide details of the radiation exposure, including a quantitative assessment of the absorbed dose, supported either by dosimetric calculations or by other information. Describe the procedures, which are in place to minimise the risks to participants and researchers. The study should also be approved by the relevant institutional Radiiation Safety authority.

Proceed to Section 12.

SECTION 12: CLINICAL TRIALS OF DRUGS OR DEVICES
(refer to the National Statement on Ethical Conduct in Research Involving Humans, p. 35-38, and also to Therapeutic Goods Administration, http://www.tga.gov.au)

This section must be completed for all applications involving clinical trial(s).

12.1 Does this section apply to your research? [x] N [ ] Y

If NO, Go to Section 13

12.2 (i) Is the research being conducted under the Clinical Trial Notification Scheme (CTN)? [ ] N [ ] Y

(ii) Is the research being conducted under the Clinical Trial Exemption Scheme (CTX)? [ ] N [ ] Y

(iii) Is the research using only approved drug(s)/device(s) in accordance with Therapeutic Goods Administration Approved Product Information? [ ] Y [ ] N
(Note reversed order of the responses)

12.3 (a) Will this research be undertaken on behalf of (or at the request of) a pharmaceutical company, or other commercial entity, or any other sponsor? [ ] N [ ] Y

If you answered YES, provide details of the name of the sponsor (and co-sponsors if any) ?
This information should be included in the Participant Information Statement and Consent Form.

Will the sponsor(s) provide any support in money or kind?
Provide details.

(b) If you answered YES to (a) will that entity undertake in writing to abide by either the Medicines Australia Guidelines for Injury Resulting from Participation in an Industry-Sponsored Clinical Trial (www.medicinesaustralia.com.au) or the ABPI Clinical Trial Compensation Guidelines? [ ] Y [ ] N

If you answered NO to this question, provide details.

(c) If you answered YES to (a), will that entity undertake in writing to indemnify the institution, the HREC(s) and the researchers ? [ ] Y [ ] N
(If you answered YES, a copy of the appropriate deed or letter of indemnity should be included with the application).

If you answered NO to this question, provide details.

(d) **If you answered YES to (a), (b) or (c), does the sponsor hold a current insurance policy to cover this project?** ☐ Y ☐ N
(If you answered YES, provide a certificate of currency).

If you answered NO to this question, provide details.

12.4 List any drugs or devices to be used, and their TGA approval status both in Australia and overseas ☐ NA

12.5 How many participants are projected to be enrolled into the trial at this site and in total? (Please give a single figure for each, not a range)

12.6 What is the projected duration of the trial, from first enrolment to the last protocol interaction with the last enrolled subject (in years)?

12.7 If all projected participants complete the protocol:

(a) **what total payment will be received from the sponsoring company?**
(Please give a single figure, not a range)

(b) **what additional "in kind" support (ie free drug, equipment, etc), if any, will be provided by the sponsoring company?**

For instructions on how to obtain TGA approval, please refer to **http://www.tga.gov.au**.

Proceed to the Section 13.

SECTION 13. DECLARATION OF RESEARCHERS

I/we apply for approval to conduct the research. If approval is granted, it will be undertaken in accordance with this application and other relevant laws, regulations and guidelines.

Name of project: Development of a novel intervention for stepping training ability to reduce risk of falls in older adults

Signature of Chief Investigator or Supervisor

Name Dr Stuart Smith (print) Signature: [signature] Date: 25/11/08

Signature of Associate Researcher(s) or Student(s)

Name Dr Stephen Lord (print) Signature: S R Lord Date: 24/11/08

Name Dr Catherine Sherrington (print) Signature: [signature] Date: 24/11/08

Name Professor Stephanie Studenski (print) Signature: ATTACHED Date: ....

SECTION 13. DECLARATION OF RESEARCHERS

I/we apply for approval to conduct the research. If approval is granted, it will be undertaken in accordance with this application and other relevant laws, regulations and guidelines.

Name of project: Development of a novel intervention for stepping training ability to reduce risk of falls in older adults

Signature of Chief Investigator or Supervisor

Name Professor Stephen Lord
(print)

Signature: .... Date: ....

Signature of Associate Researcher(s) or Student(s)

Name Dr Stuart Smith
(print)

Signature: .... Date: ....

Name Dr Catherine Sherrington
(print)

Signature: .... Date: ....

Name Professor Stephanie Studenski
(print)

Signature: [signature] Date: 4/11/08

Name ....
(print)

Signature: .... Date: ....

Signature of appropriate senior officer NOT ASSOCIATED with the research (e.g. Head of School/ Department/Unit/Dean of Faculty or Head of Division).

After careful consideration and appropriate consultation, I have reviewed the attached HREC application, including the Participant Information Statement and Consent Form. I am satisfied that the scientific merit of this work justifies its being performed and that the information which will be obtained justifies the inconvenience and risks to participants.

Name: ....
(print)

Title: ....
(print)

Position: ....
(print)

Signature: .... Date: ....

Signature of appropriate senior officer NOT ASSOCIATED with the research (e.g. Head of School/ Department/Unit/Dean of Faculty or Head of Division).

After careful consideration and appropriate consultation, I have reviewed the attached HREC application, including the Participant Information Statement and Consent Form. I am satisfied that the scientific merit of this work justifies its being performed and that the information which will be obtained justifies the inconvenience and risks to participants.

Name: **Professor Peter R Schofield** (print)

Executive Director and CEO

Title: **Prince of Wales Medical Research Institute** (print)

Position: .... (print)

Signature: [signature] Date: 25/11/08

Requested amendment to research study “Development of a novel interventions for stepping training ability to reduce risk of falls in older adults” - HREC 08351

We would like to extend our recruitment strategy. For logistical reasons and to draw a more homogenous sample, we plan to recruit our participants from independent living units (ILU) of retirement villages in the Sydney region. We have already made contact with the Anglican Retirement Villages and they are willing to support us with the recruitment and by offering us work space where we can administer our tests under standardised conditions.

Formal permission has been obtained from the Anglican Retirement Village Woolaware Shores to carry out this study on its premises. Please see attached letter.

For the recruitment of participants within the retirement village a flyer has been designed. It is proposed to mail these to the residents. Please find a copy attached.

Additional aims of intervention:

- To establish the effect of stepping exercise on falls and faller status determined by effect sizes
- To investigate the effects of stepping exercise on cognitive performance in older people

Standard pen and paper-based tests of cognition (e.g. Digit Symbol Substitution Test, Trailmaking Test, Stroop Test) will be used to measure the cognitive status of the participants before and after the intervention. In addition newly developed stepping tests of combined cognitive and motor function will be used.

One PhD student will also be involved in this project. Name: Daniel Schoene; Falls and Balance Research Group, Neuroscience Research Australia; Student ID 3337872, School of Public Health and Community Medicine, UNSW.
